# Beyond a solvent: HFIP as a game-changing medium in heterocyclic ring construction

**DOI:** 10.3762/bjoc.22.116

**Published:** 2026-10-14

**Authors:** Laxman M Alakonda, Ravi Varala, Vittal Seema, Mohammed Mujahid Alam

**Affiliations:** 1 School of Pharmacy, College of Health Sciences, University of Wyoming, Laramie, WY 82071, USA; 2 Scrips Pharma, Mallapur, Hyderabad 500076, Telangana, India; 3 Research Fellow in Health Sciences, INTI International University, 71800 Nilai, Malaysia; 4 Research Fellow, Shinawatra University, Pathum Thani 12160, Thailand; 5 Department of Chemistry, Rajiv Gandhi University of Knowledge Technologies (RGUKT), Basar, Nirmal-504107, Telangana, India; 6 Department of Chemistry, College of Science, King Khalid University, Abha, 61421, Saudi Arabia

**Keywords:** additive, catalyst, heterocycles, HFIP (hexafluoroisopropanol), reaction medium

## Abstract

1,1,1,3,3,3-Hexafluoroisopropanol (HFIP) has emerged as one of the most powerful and versatile solvents in modern organic synthesis owing to its exceptional hydrogen-bond-donating ability, low nucleophilicity, high ionizing power, and remarkable capacity to stabilize ionic and radical intermediates. These unique physicochemical properties enable HFIP to facilitate proton transfer, activate otherwise unreactive bonds, and promote a wide range of intermolecular interactions, making it highly valuable in heterocycle synthesis. Over the past decade, the application of HFIP has expanded significantly, particularly in C–H functionalization and heterocyclic ring construction, where it frequently acts not only as a solvent but also as a promoter, cocatalyst, and reaction-directing medium. In this review, we comprehensively and critically summarize the latest representative advances in HFIP-enabled heterocyclic ring formation reported from 2022 to 2025. Particular emphasis is placed on mechanistic understanding, synthetic efficiency, substrate scope, and the multifunctional role of HFIP in controlling chemo-, regio-, and stereoselectivity. HFIP-mediated transformations often proceed under mild, metal-free, oxidant-free, or redox-neutral conditions and enable challenging cyclizations, annulations, dearomatizations, rearrangements, and cross-dehydrogenative coupling reactions that are difficult to achieve using conventional solvents. Mechanistic discussions throughout the review highlight the ability of HFIP to stabilize cationic, radical, and transition-state intermediates through extensive hydrogen-bonding networks and cooperative interactions with catalysts or reagents. For clarity, the review is organized according to the structural classes of the target heterocycles and the nature of the synthetic strategies employed. The review focuses on the construction of simple five-membered heterocycles; fused heterocyclic systems, including five–six- and six–six-fused frameworks and polycyclic fused architectures; spiro heterocycles, including five–five- and five–six-spiro systems generated through radical, ionic, electrochemical, and dearomative cascade processes; and saturated and non-aromatic heterocycles, encompassing three-, five-, six-, seven-, and nine-membered rings, mixed five-/six-membered frameworks, and polyfused saturated systems. Overall, this review demonstrates that HFIP has evolved far beyond a conventional fluorinated solvent and now serves as a powerful reaction-enabling platform in modern heterocyclic chemistry. By critically comparing recent methodologies and mechanistic paradigms, this review aims to provide a unified perspective on the expanding role of HFIP and inspire future developments in sustainable heterocycle synthesis.

## Introduction

Fluorinated solvents have attracted increasing attention in recent years as specialized reaction media and co-solvents [1]. Among them, fluorinated alcohols exhibit distinctive physicochemical properties, including strong hydrogen-bond-donating ability, high polarity, and, depending on their molecular structure, varying degrees of water miscibility and volatility [2–4]. In particular, 1,1,1,3,3,3-hexafluoro-2-propanol (HFIP) is widely used in organic synthesis and allows facile product isolation [5]. Its high polarity and strong hydrogen-bond-donating ability promote the formation and stabilization of cationic intermediates, facilitating their reaction with nucleophiles [6]. The electron-withdrawing –CF_3_ groups also increase the acidity of the hydroxy proton, while fluorinated alcohols are weak nucleophiles and poor hydrogen-bond acceptors. Consequently, they exhibit reduced self-association compared with conventional alcohols [7].

**Figure 1:**
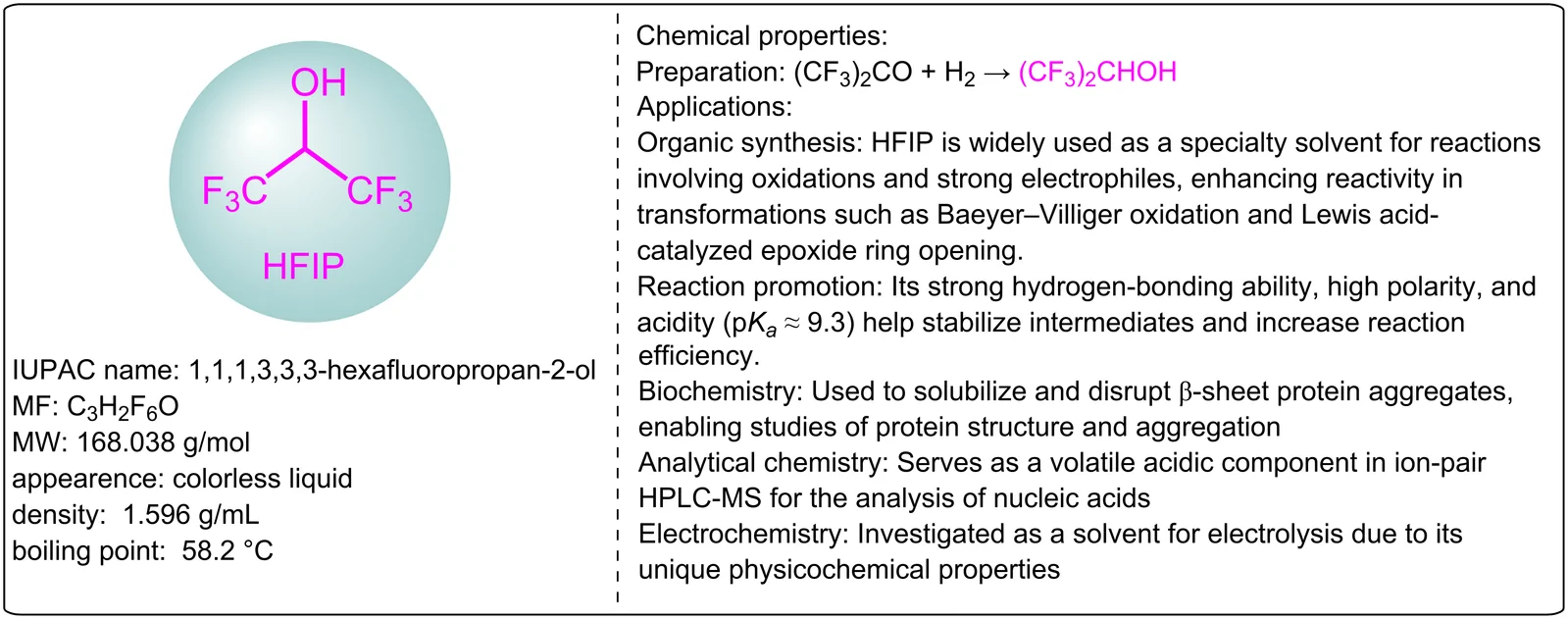
Structural features, preparation, and applications of HFIP.

Figure 1 summarizes the key physicochemical properties and diverse applications of HFIP. HFIP (C_3_H_2_F_6_O; MW 168.038 g·mol^−1^) is a colorless, highly volatile liquid (density 1.596 g·mL^−1^; bp 58.2 °C) with strong hydrogen-bond-donating ability, high polarity, and moderate acidity (p*K*_a_ ≈ 9.3). These properties make HFIP a useful specialty solvent and reaction-promoting medium in organic synthesis, as well as in biochemistry, analytical chemistry, and electrochemistry. It can also stabilize reactive intermediates and enhance reaction efficiency in various transformations.

A key advantage of HFIP and related fluorinated alcohols is their ability to promote reactions that would otherwise require catalysts or Lewis acids [8]. HFIP is a strong hydrogen-bond donor, as demonstrated by calorimetric and spectroscopic studies, as well as by the formation of stable complexes with various nucleophiles. With a boiling point of 58 °C, HFIP combines strong hydrogen-bond-donating ability, high ionizing power, moderate acidity (p*K*_a_ ≈ 9.3), and relatively low nucleophilicity [9]. Its strong hydrogen bonding with carbonyl groups can activate C=O bonds and enhance their electrophilic character. These properties arise from its high hydrogen-bond donor strength (α = 1.96), polarity (Ps = 11.08), and ionizing ability (YOTs = 3.79).

Owing to its unique combination of physicochemical properties, HFIP is often described as a “magic solvent” and has been widely applied across organic synthesis [10–21], photochemistry [22], electrochemistry [23], etc. Its use in organic synthesis has increased rapidly in recent years and continues to grow. HFIP exhibits several distinctive features, including strong hydrogen-bond donating ability, high polarity with low nucleophilicity, significant Brønsted acidity, excellent ion (particularly carbocation) stabilizing capability, strong solvation power, redox stability, and ease of recovery and recyclability. Collectively, these properties enable efficient synthetic transformations, often under catalyst-free and mild reaction conditions.

### Environmental, toxicological, and safety considerations of HFIP

Despite its useful properties, HFIP should not be considered inherently a green solvent. Its highly fluorinated nature raises environmental concerns related to solvent production, persistence, waste management, and disposal. HFIP is also volatile and requires appropriate handling to minimize exposure, including the use of a fume hood and suitable personal protective equipment. Fluorinated solvent waste should be collected and disposed of according to applicable institutional and regulatory requirements.

Therefore, the sustainability of HFIP-based processes should be evaluated beyond reaction efficiency, considering solvent quantity, recovery and recycling, energy use, and waste generation. While HFIP can offer significant advantages in reactivity and selectivity, these benefits should be balanced against its environmental and safety considerations.

The growing significance of HFIP has also been reflected in several comprehensive reviews. Solyev summarized HFIP-mediated C–H functionalization strategies [24], while Aubé and co-workers provided an extensive overview of its applications in organic synthesis [25]. More recently, Gao and co-workers updated the progress in homogeneous catalytic reactions involving HFIP [26].

## Review

### The role of HFIP in the heterocyclic ring formations

#### Five-membered heterocyclic rings

**Scheme 1:**
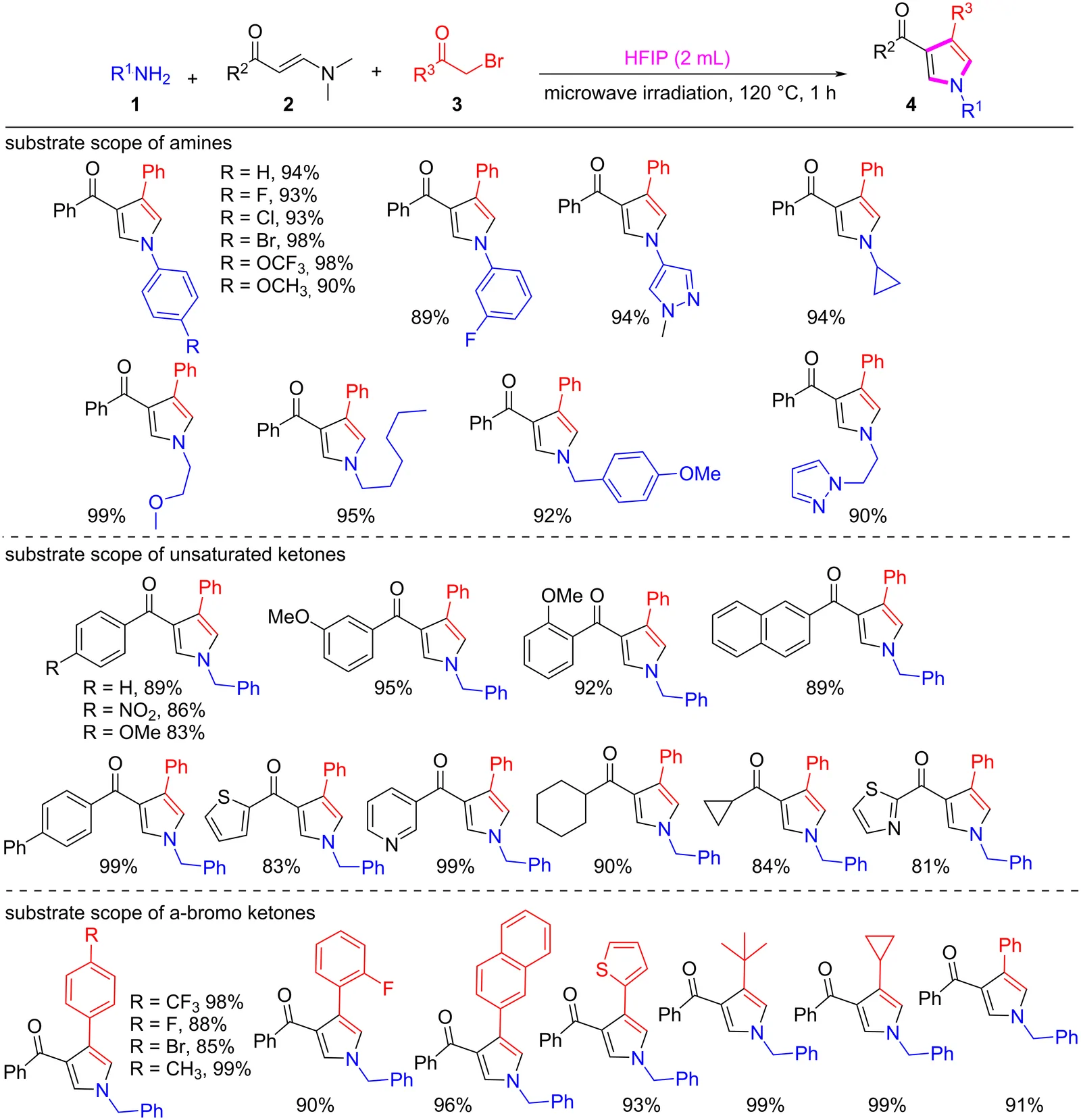
Synthesis of 3-acyl-1,4-diaryl/alkyl pyrroles **4**.

**Synthesis of 3-acyl-1,4-diaryl/alkylpyrroles:** A catalyst-free, microwave-assisted multicomponent strategy has been developed by Siddaiah and co-workers for the synthesis of 1,3,4-trisubstituted pyrroles **4** from 3-(dimethylamino)propenones **2**, amines **1**, and α-bromoketones **3** using recyclable HFIP [27] (Scheme 1). This protocol provides a simple and efficient approach, affording the desired products in 1 h with yields ranging from 52% to 99%. A key advantage of this methodology is the use of HFIP as a reusable reaction medium under microwave irradiation, enabling a catalyst-free multicomponent transformation for the efficient synthesis of 3-acyl-1,4-di(hetero)aryl/(cyclo)alkylpyrroles.

The substrate scope demonstrates broad functional-group tolerance across all three reaction components (Scheme 1). Aromatic amines bearing either electron-withdrawing or electron-donating substituents generally afforded good to excellent yields, although *ortho*-substituted fluoroaniline showed diminished reactivity, likely due to steric effects. In contrast, heteroaryl, cycloalkyl, and aliphatic amines consistently produced pyrroles in high yields, underscoring the versatility of the protocol. Similarly, diverse 3-(dimethylamino)propenones and α-bromoketones containing electron-rich, electron-deficient, heteroaryl, alkyl, and cycloalkyl substituents were well tolerated, furnishing polysubstituted pyrroles in excellent yields, often approaching quantitative conversion. Notably, substituent position on the aromatic ring exerted minimal influence on the reaction outcome, further supporting the robustness of the method.

**Scheme 2:**
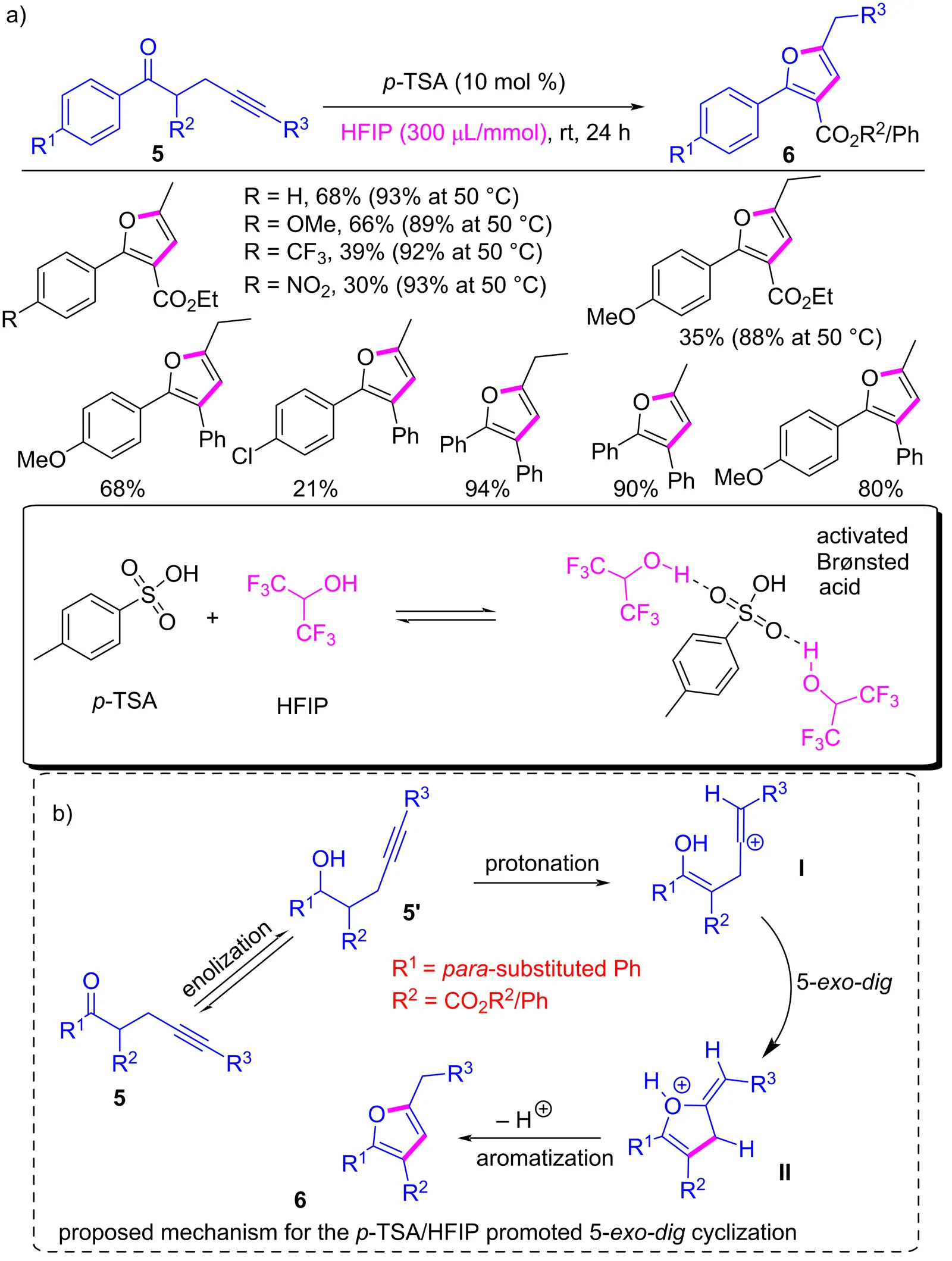
Substrate scope for the synthesis of furan derivatives and proposed mechanism.

**Synthesis of furan derivatives**: A mild metal-free protocol employing *p*-TSA (*para*-toluenesulfonic acid) in HFIP has been developed by Nguyen and Pu for the cyclization of ynones **5** into 2-aryl-3-carboxylate ester furans and 2,3-diarylfurans **6** (Scheme 2a) [28]. This methodology proceeds under relatively mild conditions and affords the desired products in good to excellent yields (up to 94%). The operational simplicity of the protocol is notable, as reactions generally produced clean mixtures with minimal byproducts and required no extensive workup prior to purification.

Substrate scope studies revealed broad tolerance toward structurally diverse ynones, although substrate electronics strongly influenced reaction efficiency. The substrate scope revealed a dependence of the reaction yield on the electronic nature of the substituents; however, no consistent correlation between electron-rich or electron-deficient substrates and higher yields was observed. In particular, α-carboxyloxy ynones initially exhibited lower conversions under room-temperature conditions, but increasing either the reaction time or temperature substantially improved yields, demonstrating the adaptability of the method. In contrast, electron-rich 2,3-diarylfurans could be synthesized efficiently even under ambient conditions (Scheme 2a).

Mechanistically, HFIP appears to play a dual role as a highly ionizing, hydrogen-bond-donating solvent and as an activator of *p*-TSA, effectively enhancing its Brønsted acidity. The reaction is proposed to proceed through protonation of the enolized ynone, followed by formation of a carbocation intermediate **I** and subsequent 5-*exo-dig* cyclization **II** to furnish the furan core after aromatization (Scheme 2b).

**Scheme 3:**
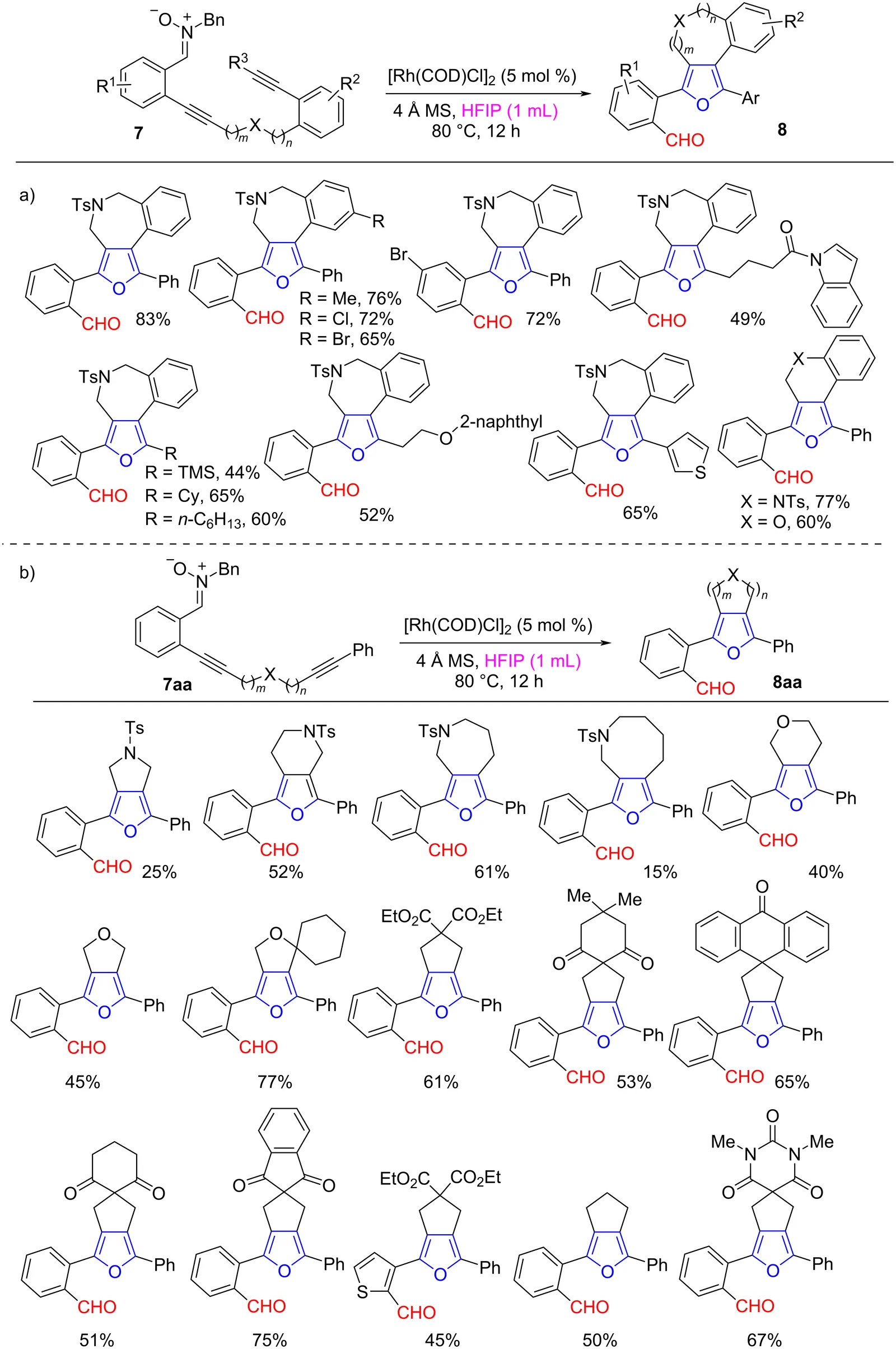
a) Rhodium(I)-catalyzed cascade annulation of benzene-tethered 1,*n*-diynyl nitrones. b) Rhodium(I)-catalyzed cascade annulation of alkyl-tethered 1,*n*-diynyl nitrones.

**Synthesis of 3,4-fused fully substituted furan derivatives**: Nitrones are versatile synthetic intermediates widely employed in the rapid assembly of structurally complex molecules relevant to pharmaceutical and materials chemistry [29]. In this context, a Rh(I)-catalyzed and HFIP-assisted cascade annulation of 1,*n*-diynyl nitrones **7** has been developed by Rao and co-workers for the synthesis of fully substituted furans **8** fused to diverse 5–8 membered carbocycles, heterocycles, and spirocycles (Scheme 3a) [30].

The transformation proceeds through a complex cascade sequence involving Rh(I)-mediated oxycyclization, N–O bond cleavage, and generation of an α-oxo rhodium carbene intermediate, followed by carbene-assisted migration (CAM), heterocyclization, and hydrolysis. Mechanistic studies suggest that HFIP plays a crucial role through hydrogen-bonding interactions with organorhodium intermediates, suppressing competing azomethine ylide pathways and instead promoting an unprecedented CAM/heterocyclization sequence leading to fused furan frameworks. This highlights the unique ability of HFIP to modulate organometallic reactivity beyond its conventional solvent role. This cascade annulation offers a highly modular and structurally diverse route to fused and spirocyclic furan architectures in a single operation. The combination of Rh(I) catalysis and HFIP-assisted pathway control enables access to reactivity patterns that are otherwise difficult to achieve, while the scalability and downstream functionalization of the products further underscore the synthetic utility of the methodology.

The methodology demonstrates a broad substrate scope and accommodates a wide range of tether architectures and functional groups (Scheme 3b). N- and O-tethered diynyl nitrones **7aa** efficiently furnish fused furans **8aa** containing zepine, chromene, pyridine, pyran, tetrahydrofuran, cyclopentene, and spirocyclic motifs in moderate to good yields. Substrates bearing alkyl, cycloalkyl, heteroaryl, sulfone, sulfonamide, ester, hydroxy, and ferrocenyl substituents are all well tolerated. In contrast, terminal alkynes and larger ring-forming substrates such as certain 1,9-diynyl systems provide diminished yields, likely due to unfavorable entropic and transannular effects during cyclization. The protocol is also applicable to late-stage functionalization, as demonstrated by annulation of estrone-derived substrates to generate structurally complex furanyl derivatives.

**Scheme 4:**
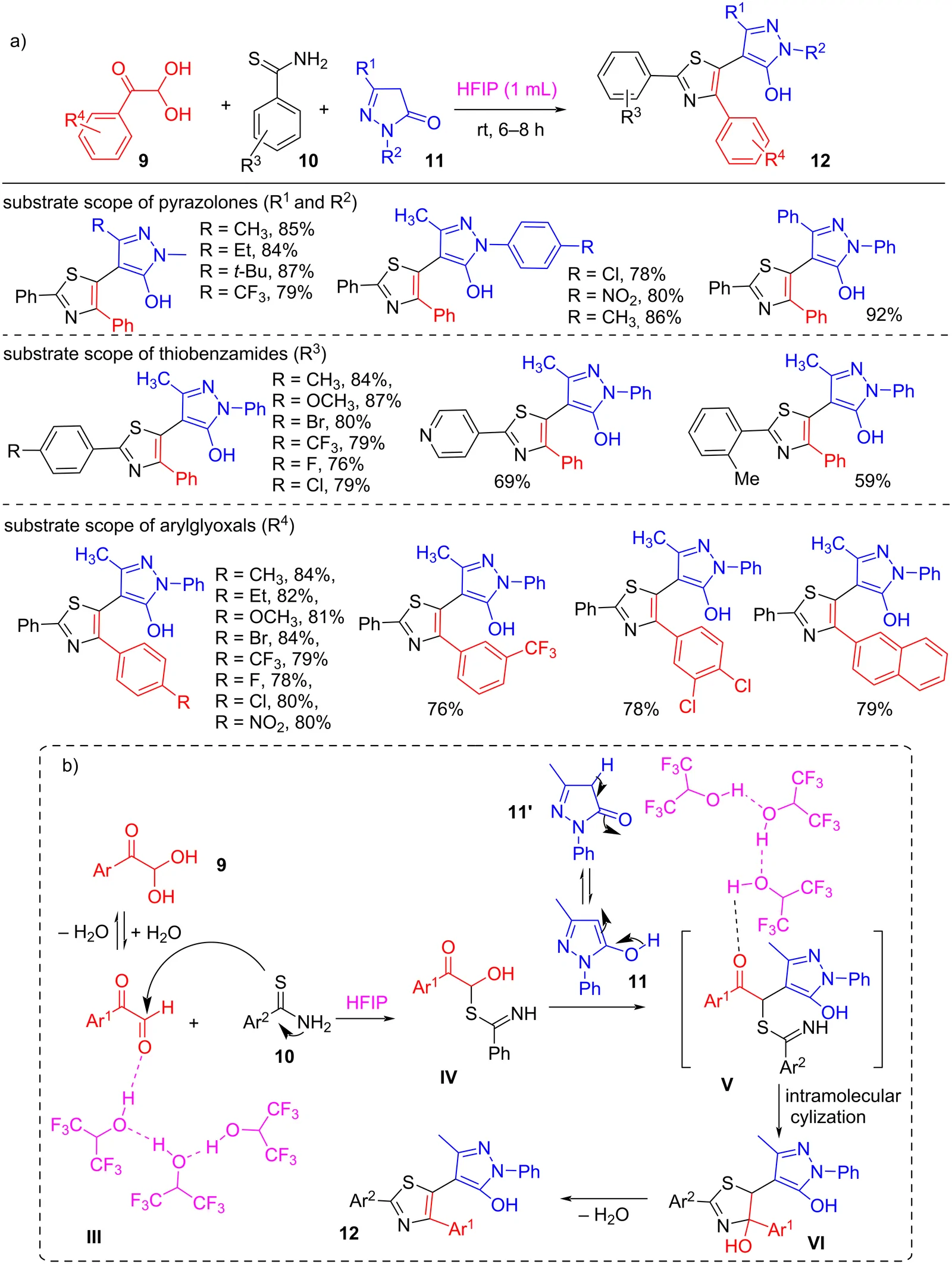
a) Substrate scope of pyrazolones. b) Proposed mechanism for the products **12**.

**Synthesis of pyrazole-linked thiazole derivatives:** Thiazoles are important sulfur- and nitrogen-containing heterocycles widely encountered in natural products, pharmaceuticals, and functional materials [31]. In this context, a green multicomponent protocol has been developed by Choudhury and co-workers for the synthesis of pyrazole-linked thiazoles **12** using arylglyoxals **9**, aryl thioamides **10**, and pyrazolones **11** in HFIP under room temperature and additive-free conditions (Scheme 4a) [32]. Compared with conventional thiazole syntheses that often require elevated temperatures, catalysts, or multistep procedures, this methodology proceeds under remarkably mild conditions while efficiently constructing multiple C–C, C–N, and C–S bonds in a single operation. The protocol also demonstrates broad applicability, furnishing structurally diverse thiazole-linked pyrazoles **12** in good to excellent yields, and can be extended to the synthesis of thiazole-linked barbiturates and imidazole-linked pyrazoles **12**. The present approach offers several advantages from a green chemistry perspective, including room temperature operation, metal- and additive-free conditions, high atom economy, water as the sole byproduct, solvent recyclability, and operational simplicity. The absence of chromatographic purification further enhances its practicality and scalability.

Substrate scope investigations revealed good tolerance toward electronically diverse pyrazolones, aryl thioamides, and arylglyoxals. Both electron-donating and electron-withdrawing substituents on thioamides and arylglyoxals generally afforded the desired products in high yields, indicating the robustness of the transformation. However, pyridyl and *ortho*-substituted thioamides showed comparatively lower reactivity, likely due to electronic and steric effects, respectively, whereas aliphatic thioamides were ineffective under the optimized conditions. In contrast, substituent variation on arylglyoxals had minimal influence on product yields, and even aliphatic glyoxals were compatible, further highlighting the versatility of the protocol (Scheme 4a).

Mechanistically, HFIP is proposed to play a crucial role by activating the glyoxal substrate through hydrogen bonding **III** and facilitating formation of key intermediates prior to intramolecular cyclization **V** and dehydration **VI** to yield the product **12** (Scheme 4b).

**Scheme 5:**
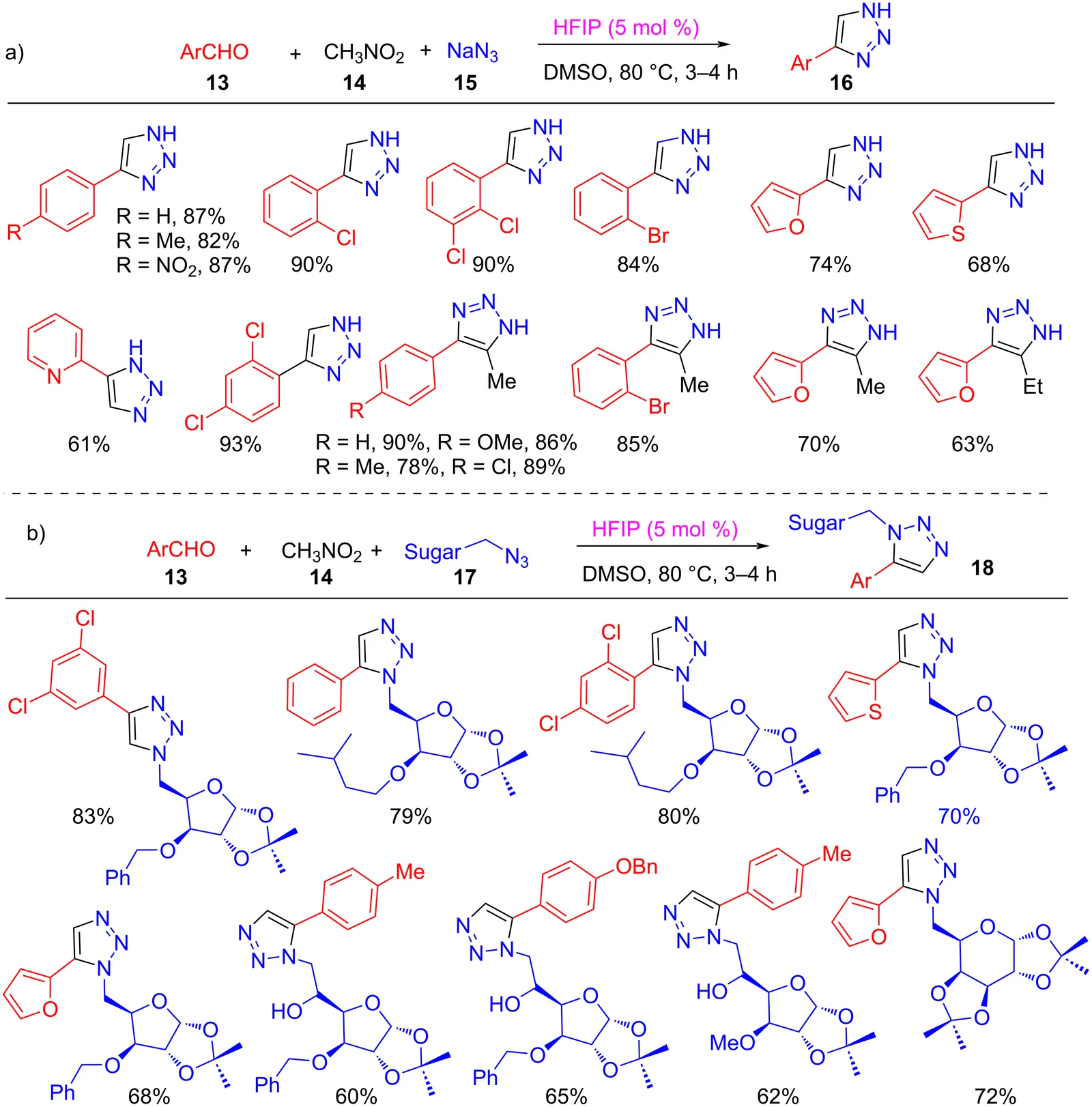
Substrate scope of the a) synthesis of *N*-unsubstituted **16** and b) *N*-substituted 1,2,3-triazoles **18**.

**Synthesis of 4-aryl-*NH*-1,2,3-triazoles and 1,5-disubstituted 1,2,3-triazolyl glycoconjugates:** 1,2,3-Triazoles constitute an important class of nitrogen-containing heterocycles widely employed in medicinal chemistry, materials science, and chemical biology because of their remarkable stability and diverse biological activities [33]. Consequently, the development of efficient, metal-free, and regioselective methods for triazole synthesis remains an active area of research. In this regard, an HFIP-promoted multicomponent strategy has been developed by Tiwari and co-workers for the synthesis of *N*-unsubstituted **16** and *N*-substituted 1,2,3-triazoles **18** from aldehydes **13**, nitromethane (**14**), and sodium azide (**15**) or glycosyl azides **17** (Scheme 5) [34]. This methodology enables sequential formation of one C–C and two C–N bonds in a single operation under metal-free conditions. The protocol proceeds with high regioselectivity, gram-scale applicability, operational simplicity, and broad functional-group tolerance while providing the desired triazoles in generally good to excellent yields. Herein, HFIP provides the required mildly acidic environment to achieve optimal triazole formation, serving as a safer alternative to hazardous hydrazoic acid while enabling high product yields under the optimized conditions.

Substrate scope studies demonstrated that aromatic aldehydes bearing electron-donating, electron-neutral, and electron-withdrawing substituents were all compatible with the reaction conditions (Scheme 5a). Halogen- and nitro-substituted substrates afforded products in particularly high yields, while *ortho*-substituted aldehydes were also well tolerated, indicating minimal steric sensitivity. Heteroaromatic aldehydes containing furan, thiophene, and pyridine motifs underwent smooth conversion to the corresponding triazoles, although aliphatic aldehydes proved unreactive under the optimized conditions. Extension of the methodology to nitroethane enabled efficient access to 4,5-disubstituted *NH*-1,2,3-triazoles **16**, with electronic variations on the aldehyde component exerting little influence on product yields.

Importantly, the strategy was further expanded to the regioselective synthesis of 1,5-disubstituted triazolyl glycoconjugates **18** using glycosyl azides **17** and aldehydes **13**, nitromethane (**14**) (Scheme 5b). Both substituted aromatic and heteroaromatic aldehydes were well tolerated, providing glycoconjugates in moderate to good yields. This extension highlights the synthetic utility of the protocol for accessing biologically relevant triazole-linked architectures.

#### Five-six fused heterocycles

**Scheme 6:**
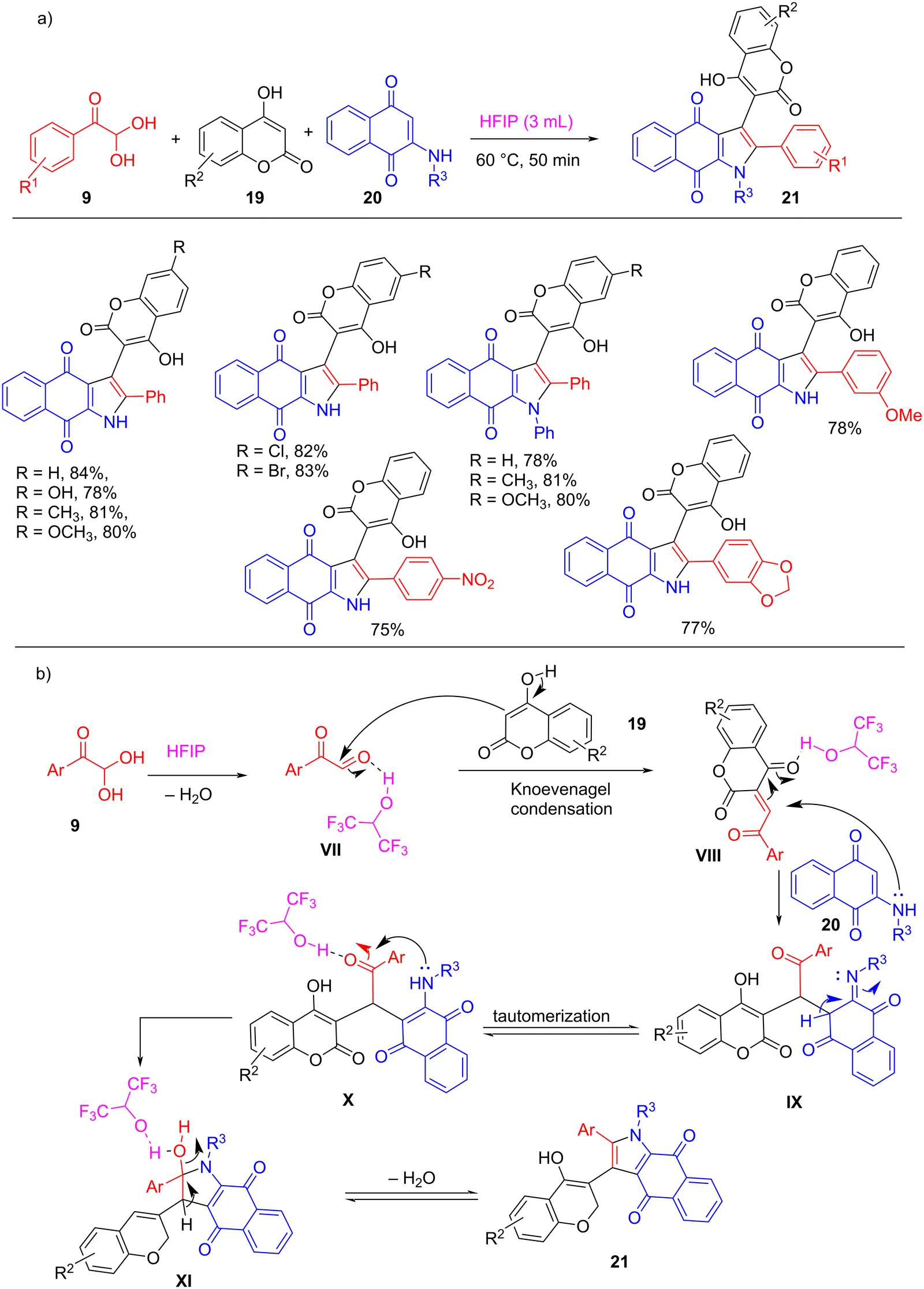
a) Substrate scope for synthesizing naphthoquinone-fused pyrrole derivatives **21**. b) Proposed mechanism.

**Synthesis of coumarin-linked naphthoquinone-fused pyrrole derivatives**: Coumarin-, pyrrole-, and naphthoquinone-based scaffolds are well known for their broad spectrum of biological and pharmacological activities, making hybrid molecules containing these motifs highly attractive targets in medicinal chemistry [35]. In this context, Chaurasia and Parvin reported a simple one-pot protocol for the synthesis of coumarin-linked pyrrole–naphthoquinone hybrids **21** via the reaction of arylglyoxals **9**, 4-hydroxycoumarins **19**, and 2-amino-1,4-naphthoquinones **20** in HFIP under reflux conditions (Scheme 6a) [36].

This method provides a catalyst-free, operationally straightforward, and efficient alternative for assembling structurally complex heterocycles. Notably, the reaction enables the formation of two C–C bonds and one C–N bond in a single operation, delivering the desired products in good yields within relatively short reaction times. Here, HFIP acts as an effective reaction medium, promoting efficient multicomponent coupling under reflux conditions and facilitating clean bond-forming events without the need for external catalysts. A key practical advantage of this protocol is its simple work-up, as the products can be purified by recrystallization rather than column chromatography, thereby enhancing both scalability and synthetic convenience.

Substrate scope studies demonstrated broad tolerance toward structurally diverse coupling partners. Various substituted 4-hydroxycoumarins bearing halogen, hydroxy, methyl, and methoxy groups underwent smooth conversion to the corresponding products in good yields. Similarly, aryl glyoxals containing either electron-donating or electron-withdrawing substituents were compatible with the reaction conditions, indicating minimal electronic sensitivity. The methodology also tolerated different *N*-substituted amino-naphthoquinones, further supporting the generality of the transformation (Scheme 6a).

Mechanistically, HFIP is proposed to activate the carbonyl functionality of arylglyoxals **9** through strong hydrogen-bonding interactions (**VII**), facilitating nucleophilic addition of 4-hydroxycoumarin **19** followed by Michael-type addition (**VIII**) of amino-naphthoquinone **20**. Subsequent tautomerization (**IX**), cyclization (**X**), and dehydration (**XI**) steps furnish the fused pyrrole products **21** (Scheme 6b).

**Scheme 7:**
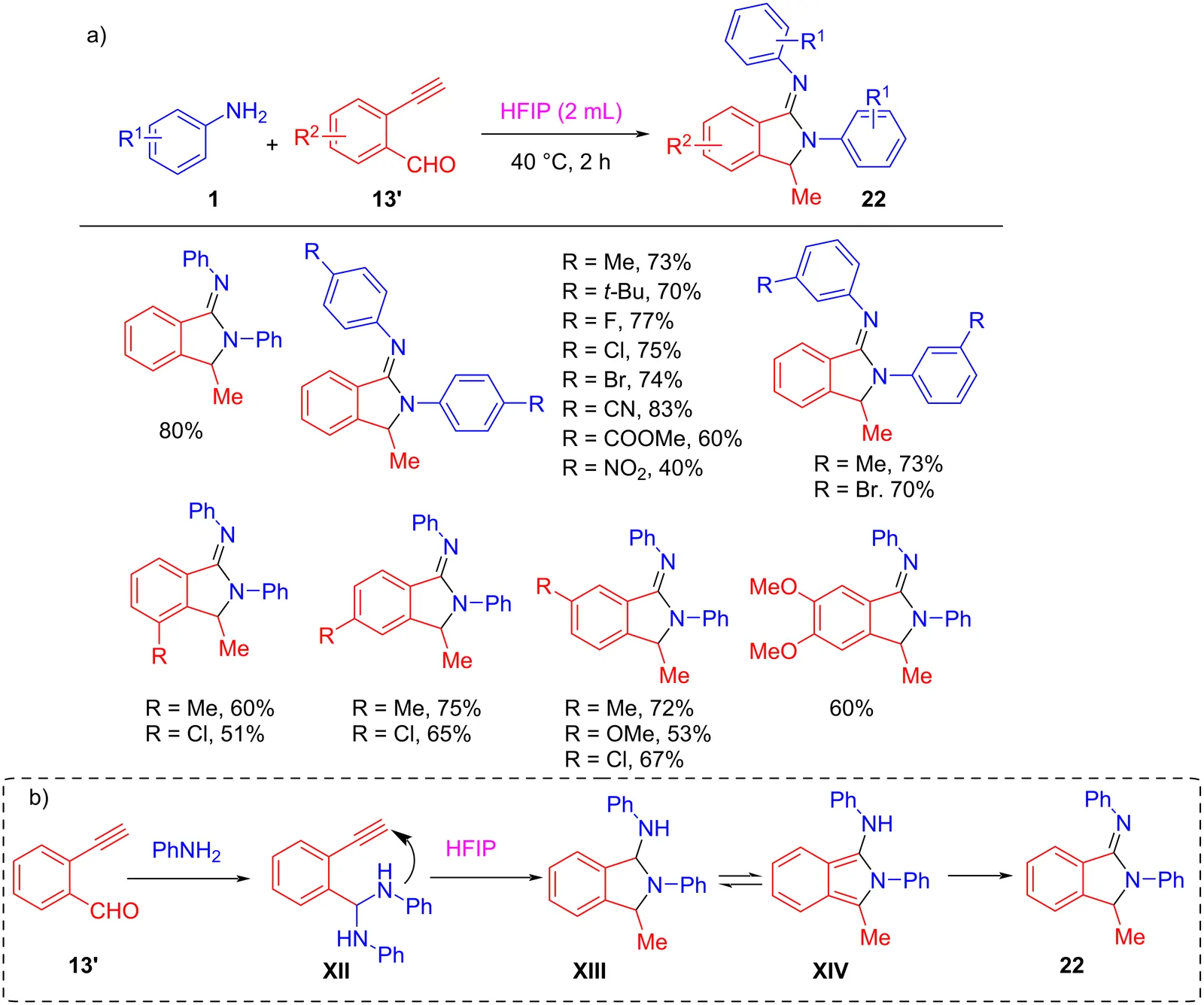
a) Substrate scope for the synthesis of **22**. b) Proposed mechanism.

**Construction of nitrogen-containing heterocycles:** Nitrogen-containing heterocycles are among the most important structural motifs in pharmaceuticals and functional materials, with a substantial proportion of FDA (Food and Drug Administration)-approved drugs incorporating at least one nitrogen heterocycle [37]. In particular, isoindolin-1-imines and related isoquinolinium salts have attracted increasing attention because of their diverse biological and synthetic utility [38]. To access these frameworks, a mild and transition-metal-free strategy has been developed by Ji and co-workers using *ortho*-ethynylbenzaldehydes **13’** and anilines **1** in HFIP (Scheme 7) [39]. This methodology offers a simpler and more sustainable route to isoindolin-1-imines **22** and isoquinolinium salts under relatively mild conditions.

The protocol demonstrated a broad substrate scope and good functional-group tolerance (Scheme 7a). Anilines bearing electron-donating substituents generally afforded the desired isoindolin-1-imines in good yields, while halogen substituents were also well tolerated, providing synthetically useful products suitable for further derivatization. Electron-withdrawing groups such as cyano substituents remained compatible, although ester- and nitro-substituted anilines exhibited reduced reactivity, likely due to decreased nucleophilicity. Meta-substituted anilines and a variety of substituted *ortho*-ethynylbenzaldehydes also participated effectively, highlighting the versatility of the transformation. Notably, steric hindrance exerted only a limited effect on reaction efficiency. However, amides, aliphatic amines, and hydroxylamine hydrochloride were ineffective under the optimized conditions, indicating certain substrate limitations.

Mechanistically, HFIP plays a central role by promoting nucleophilic addition and facilitating subsequent cyclization through its strong proton-donating and hydrogen-bonding properties. The proposed pathway involves initial aminal formation (**XII**) followed by intramolecular annulation (**XIV**) to generate the isoindolin-1-imine framework **22**. In the presence of ammonium iodide, the reaction pathway diverges toward isoquinolinium salt formation via hydroiodination and cyclization processes. Density functional theory (DFT) calculations supported the proposed mechanism and provided insight into the energetics of the key transition states (Scheme 7b).

**Scheme 8:**
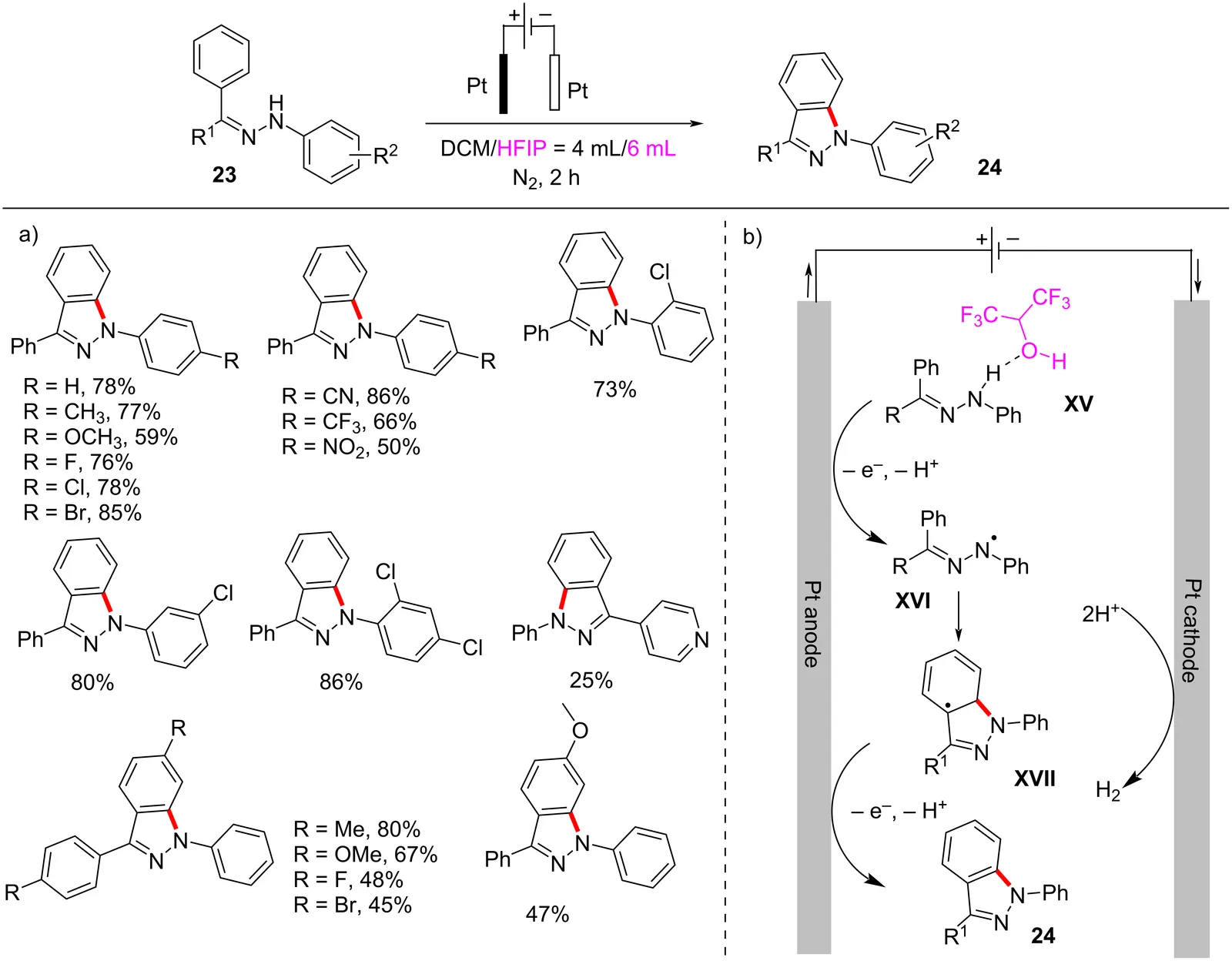
a) Substrate scope of arylhydrazones. b) Proposed mechanism for **24**.

**Synthesis of 1*H*-indazoles by an electrochemical radical C(sp^2^)–H/N–H cyclization**: An electrochemical oxidative C(sp^2^)–H/N–H cyclization strategy has been developed by Lei and co-workers for the synthesis of 1*H*-indazoles through anodic oxidation of arylhydrazones under metal- and chemical oxidant-free conditions (Scheme 8) [40]. HFIP played a dual role as solvent/proton donor and promoter of N-centered radical formation via hydrogen-bond-assisted activation. This protocol offers operational simplicity, low-cost electrode compatibility, and sustainable reaction conditions while avoiding external oxidants. The methodology exhibited broad functional-group tolerance, affording 1*H*-indazole derivatives **24** in moderate to good yields. This electrochemical protocol provides a greener and mechanistically distinct alternative, although regioselectivity issues and reduced efficiency with heterocyclic or protected substrates remain notable limitations.

Both electron-donating and electron-withdrawing substituents on the N-phenyl ring were compatible with the reaction conditions (Scheme 8a), with several electron-withdrawing substituents providing good to high yields. Halogenated substrates, including mono- and disubstituted analogues, also reacted efficiently, demonstrating the broad substrate tolerance of the protocol and the synthetic utility of halide-containing products for further derivatization.

Substituent effects on the hydrazone aryl ring significantly influenced the reaction outcome. Electron-rich disubstituted substrates delivered comparatively higher yields, whereas electron-deficient analogs afforded only moderate yields, indicating differing electronic effects depending on the substitution pattern. Monosubstituted methyl and methoxy derivatives generated regioisomeric mixtures. The detailed regioisomeric substrate scope and product distributions are not shown in Scheme 8a; readers are referred to the original report for further details. In contrast, N-protected substrates failed to undergo cyclization, highlighting the necessity of a free N–H bond for radical generation. Heterocycle-containing substrates were tolerated only in low yields, and alkene- or alkyne-substituted substrates converged to the same product. Certain substrates predominantly decomposed under the reaction conditions, reflecting limitations in substrate stability and substrate scope.

Mechanistic studies suggested that HFIP-assisted activation of arylhydrazones **23** facilitates proton-coupled electron transfer (PCET) at the anode to generate an N-centered radical intermediate **XVI**. Subsequent intramolecular radical addition (**XVII**) to the arene ring, followed by anodic oxidation and deprotonative aromatization, furnished the indazole products **24**, while cathodic proton reduction released H_2_ (Scheme 8b). Cyclic voltammetry studies further confirmed the critical role of the DCM/HFIP system in promoting N-centered radical formation.

**Scheme 9:**
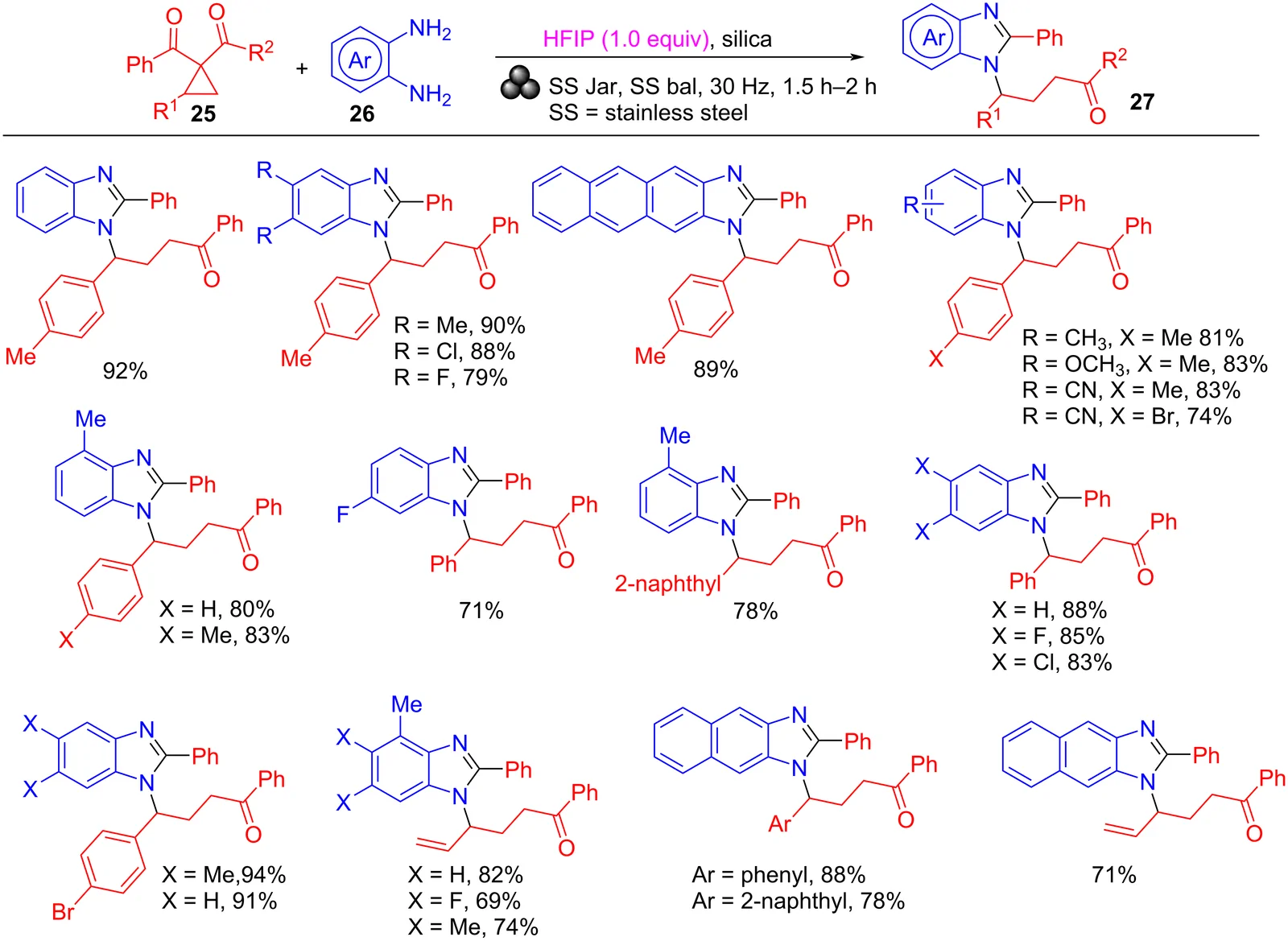
Generality of the reaction for the synthesis of compound **27**.

*Direct synthesis of 1,2-disubstituted benzimidazoles:* Benzimidazoles are important dinitrogen-containing bicyclic heterocycles widely found in pharmaceuticals, agrochemicals, and biologically active molecules [41]. A solvent-free and metal-free mechanochemical strategy has been developed by Yaragorla and co-workers for the synthesis of 1,2-disubstituted benzimidazoles **27** from donor–acceptor cyclopropyl ketones **25** and *o*-phenylenediamines **26** using stoichiometric HFIP under ambient conditions (Scheme 9) [42]. This mechanochemical approach significantly reduced reaction times while maintaining high efficiency, highlighting the advantages of mechanochemistry in sustainable heterocycle synthesis.

The protocol demonstrated a broad substrate scope with both symmetrical and unsymmetrical *o*-phenylenediamines **26**, generally affording the desired benzimidazoles **27** in good to excellent yields (Scheme 9). Symmetrical diamines generally underwent clean cyclization, whereas unsymmetrical substrates afforded regioisomeric mixtures, with the regioselectivity influenced by the electronic nature of the substituents. The detailed substrate scope, including the regioisomeric distributions, is not shown in Scheme 9; readers are referred to the original report for comprehensive details on the observed regioselectivity and product distributions.

In several cyano-substituted cases, the major regioisomers were separable and isolated in high yields. The methodology also tolerated a wide range of donor-acceptor cyclopropanes, including aryl-, vinyl-, diketone-, and keto ester-substituted systems. Notably, unsymmetrical cyclopropanes bearing both methyl ketone and aryl ketone functionalities displayed marked chemoselectivity, preferentially forming 2-methylbenzimidazoles over the corresponding 2-aryl analogues. Mechanistic studies suggested a tandem sequence involving cyclopropane ring opening, cyclization, and retro-Mannich fragmentation. Herein, HFIP acts both as the reaction solvent and as a crucial promoter of the mechanochemical transformation, as no reaction occurred in its absence, highlighting its key role in enhancing reaction efficiency.

**Scheme 10:**
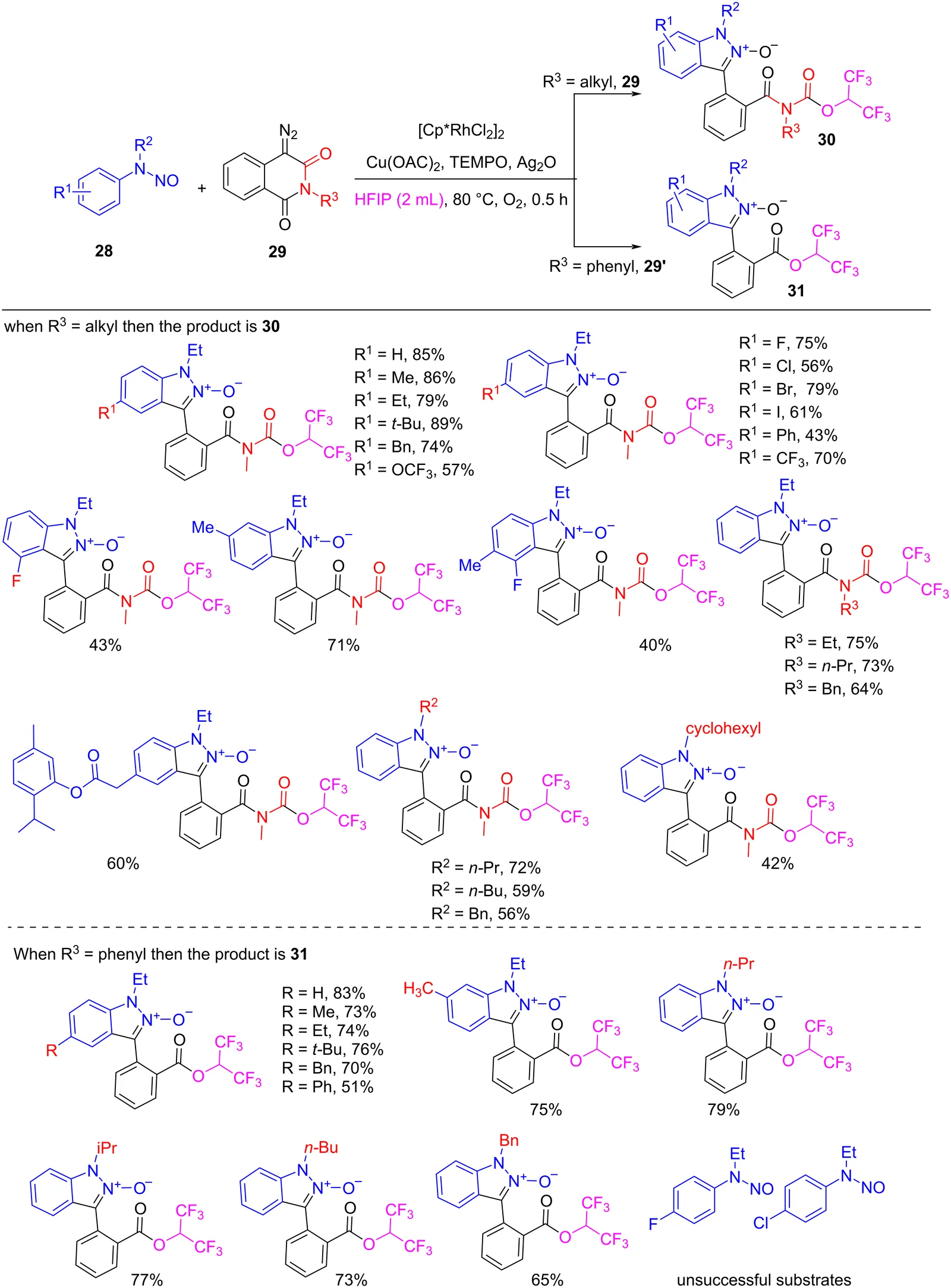
Substrate scope for the synthesis of products **30** and **31**.

*Substrate-dependent selective synthesis of diversely functionalized indazole derivatives:* Diversity-oriented synthesis (DOS) has emerged as a powerful strategy in drug discovery for rapidly accessing structurally diverse molecular libraries from simple building blocks [43]. In parallel, direct C–H functionalization has transformed modern synthesis by enabling step- and atom-economical construction of complex molecules, including pharmaceuticals and functional materials [44]. Within this context, a substrate-dependent divergent cascade approach has been developed by Fan and co-workers for the selective synthesis of functionalized indazole derivatives **30** and **31** from N-nitrosoanilines **28** and diazo compounds **29** and **29’** (Scheme 10) [45]. This strategy demonstrates how cascade C–H activation combined with substrate-dependent divergence can efficiently generate structurally distinct indazole scaffolds under mild conditions. This approach offers enhanced molecular diversity, high chemoselectivity, and operational simplicity, making it particularly valuable for diversity-oriented synthesis and early-stage drug discovery.

Notably, product divergence is controlled by the nature of the diazo coupling partner (Scheme 10): *N*-alkyl diazo homophthalimides **29** furnish benzoyl carbamate-substituted indazoles **30**, whereas *N*-phenyl analogues **29**’ lead to benzoate-functionalized indazoles **31** via an additional O-nucleophilic addition and subsequent fragmentation. This switchable reactivity highlights a key advantage of substrate-controlled selectivity in building molecular diversity from a common intermediate (Scheme 10). Herein, HFIP functions not only as the solvent but also as a key reaction participant, promoting product formation through O-nucleophilic addition to reactive intermediates. Its involvement facilitates the proposed ketal collapse, oxidative dehydrogenation, and subsequent C–C and/or C–N bond cleavage pathways, thereby contributing to the formation of the distinct products. The detailed mechanistic pathway, including the ketal collapse step, is not shown in Scheme 10; readers are referred to the original article for further mechanistic details.

A range of *N*-nitrosoanilines bearing electron-donating and electron-withdrawing groups at the *para*-position undergo smooth conversion to give products in moderate to excellent yields, while *meta*-substituted systems exhibit predictable regioselectivity governed by electronic and steric effects. Likewise, variation in alkyl substituents is well tolerated, reflecting the robustness of the cascade process. Diazo component variation is also feasible, further expanding structural diversity and enabling late-stage functionalization, including derivatization of bio-relevant motifs such as thymol.

The transformation proceeds through a cascade sequence involving nitroso-directed C–H alkylation followed by intramolecular and intermolecular nucleophilic additions, C–C or C–N bond cleavage, and dehydrogenation.

**Scheme 11:**
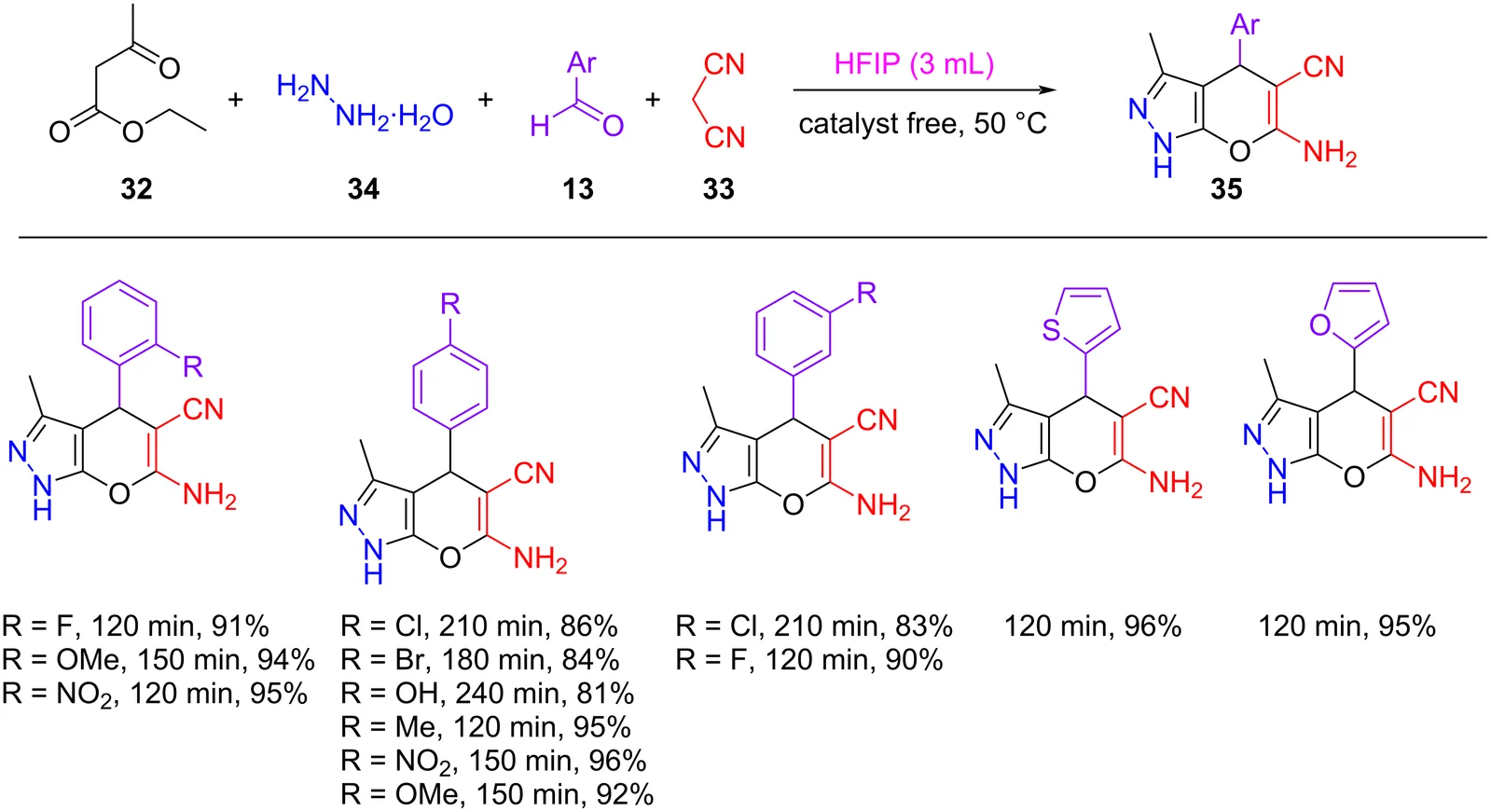
Synthesis of dihydropyrano[2,3-*c*]pyrazoles **35**.

*Synthesis of dihydropyrano[2,3-c]pyrazole scaffolds:* Dihydropyrano[2,3-*c*]pyrazoles are important heterocyclic scaffolds that exhibit a wide range of biological activities, including anticancer, antimicrobial, analgesic, molluscicidal, and kinase inhibitory properties [46]. A catalyst-free protocol has been developed by Mohamadpour for the synthesis of dihydropyrano[2,3-*c*]pyrazoles **35** using HFIP as a reusable promoting medium (Scheme 11) [47]. The transformation proceeds through a tandem Knoevenagel–Michael cyclocondensation sequence under mild conditions, enabling rapid one-pot construction of the heterocyclic framework in excellent yields. This strategy employs polyfluorinated alcohol as both solvent and promoter, highlighting the unique activating ability of HFIP in facilitating multicomponent cyclizations and provides a greener and more sustainable alternative by eliminating external catalysts, minimizing waste generation, and avoiding hazardous reagents.

The methodology demonstrates a broad substrate tolerance, with a variety of aldehyde derivatives **13** reacting efficiently irrespective of their electronic nature (Scheme 11). Product isolation is operationally straightforward, involving simple filtration and recrystallization without extensive purification procedures.

**Scheme 12:**
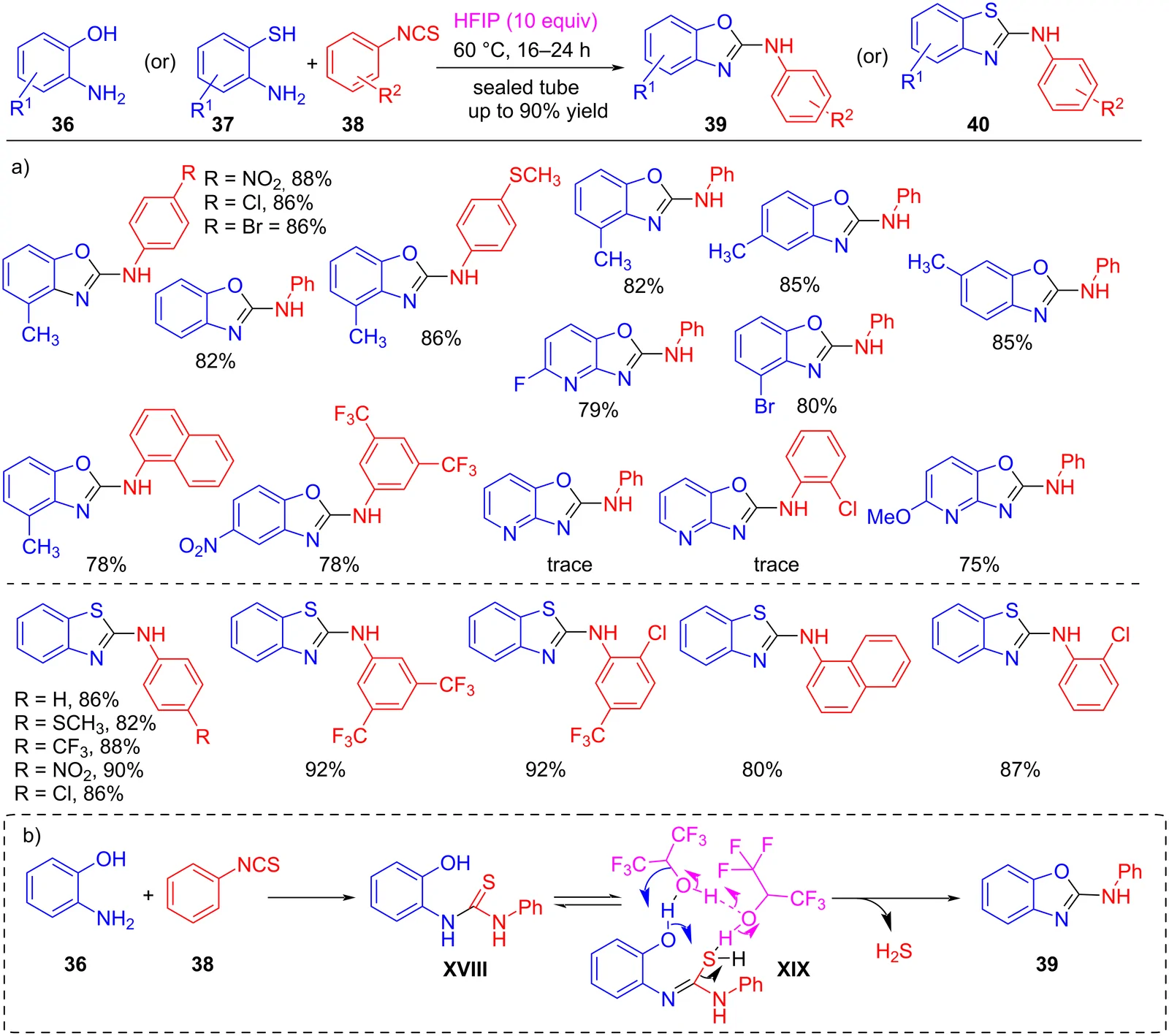
a) Substrate scope for the synthesis of **39** and **40**. b) Proposed mechanism.

*Synthesis of 2-aminobenzoxazole and 2-aminobenzothiazole derivatives:* Azole derivatives such as benzoxazoles **39** and benzothiazoles **40** are important pharmacophores widely encountered in biologically active molecules and therapeutic agents, making their efficient synthesis a central objective in heterocyclic chemistry. In this context [48], a straightforward HFIP-promoted cyclodesulfurization strategy has been developed by Malakar and co-workers for the synthesis of pharmaceutically relevant 2-aminobenzoxazoles **39** and 2-aminobenzothiazoles **40** from 2-aminophenols **36**, 2-aminothiophenols **37**, and functionalized phenyl isothiocyanates **38** (Scheme 12a).

The methodology exhibits a broad substrate scope and tolerates a wide range of electron-donating and electron-withdrawing substituents on both aminophenol and isothiocyanate partners (Scheme 12a). In general, electron-rich aminophenols afforded slightly higher yields than electron-deficient analogues, whereas electron-poor isothiocyanates performed better due to enhanced electrophilicity of the isothiocyanate carbon center, which facilitates thiourea formation and subsequent cyclization. Notably, benzothiazole **40** formation generally proceeded more efficiently than benzoxazole synthesis, likely reflecting the greater nucleophilicity of sulfur relative to oxygen. However, heteroaryl substrates such as 2-amino-3-hydroxypyridine were reported to be incompatible with the reaction conditions. This example is not shown in the scheme; readers are referred to the original article for further details. The incompatibility was attributed to the decreased nucleophilicity of the amino group due to the electron-withdrawing effect of the ring nitrogen, which may hinder intermediate formation (Scheme 12a).

The transformation proceeds under metal-, base-, and oxidant-free conditions at moderate temperature, with HFIP serving as both solvent and promoter. Mechanistically, the process involves initial thiourea formation (**XVIII**) followed by HFIP-assisted cyclodesulfurization of interemediate **XIX** through hydrogen-bond activation of hydroxy or thiol groups, ultimately leading to heterocycle formation **39** with concomitant release of H₂S (Scheme 12b). The dual role of HFIP as a proton donor and activating medium is crucial for facilitating bond cleavage and cyclization under mild conditions.

#### Construction of six-six fused heterocycles

**Scheme 13:**
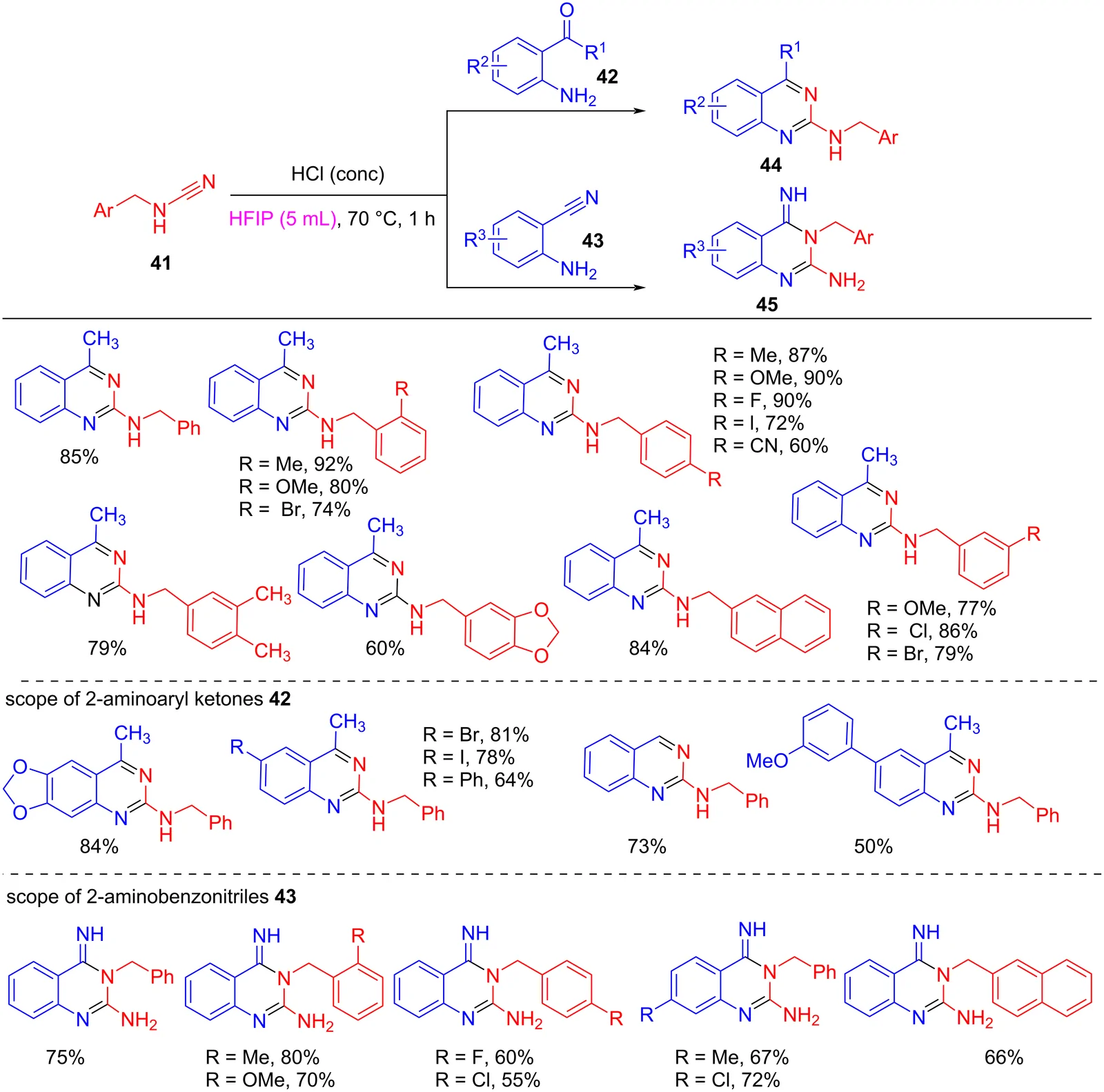
Synthesis of 2-aminoquinazoline derivatives **44** and **45**.

**Synthesis of 2-aminoquinazoline derivatives:** Quinazoline derivatives are important nitrogen-containing heteroaromatic scaffolds widely recognized for their diverse biological and pharmaceutical activities [49]. Among them, 2-aminoquinazolines constitute a prominent class of bioactive molecules and medicinally relevant intermediates. In this context, an efficient hydrochloric acid-mediated [4 + 2] annulation strategy has been developed by Wu and co-workers for the synthesis of 2-aminoquinazolines **44** from *N*-benzyl cyanamides **41** and 2-aminoaryl ketones **42** (Scheme 13) [50]. This acid-mediated annulation provides a concise and operationally simple route to two distinct classes of quinazoline derivatives. The broad substrate tolerance, high efficiency, and divergent product formation underscore the practicality and synthetic utility of this methodology for the preparation of pharmaceutically relevant quinazoline frameworks. Replacing the ketone substrates with 2-aminobenzonitriles **43** enabled direct access to 2-amino-4-iminoquinazolines **45**, thereby expanding the synthetic versatility of the protocol*.* Herein, HFIP functions as the reaction solvent, while MsOH was identified as an effective acid additive during the optimization studies. Under the reported reaction conditions, HCl was used as the acid to facilitate the overall transformation.

The methodology demonstrates a broad substrate scope and excellent functional-group tolerance (Scheme 13). *N*-Benzyl cyanamides **41** bearing electron-donating, electron-withdrawing, halogen, and heteroaromatic substituents undergo smooth annulation to furnish the desired quinazoline products in generally good to excellent yields. Notably, sterically demanding substrates such as 2-naphthyl derivatives remain compatible, indicating the robustness of the transformation. Similarly, various substituted 2-aminoaryl ketones **42**, including electron-rich, electron-poor, and halogenated systems, participate effectively in the cyclization process. Aldehyde- and diaryl ketone-derived substrates are also well tolerated, further highlighting the flexibility of the reaction design. An interesting divergence in reactivity was observed when 2-aminobenzonitriles **43** were employed instead of 2-aminoaryl ketones **42**, leading selectively to 2-amino-4-iminoquinazolines **45**. This alternative pathway also accommodates a diverse range of cyanamide and aminobenzonitrile substrates, including electronically varied and sterically hindered derivatives, generally affording moderate to high yields.

#### Construction of polycyclic fused heterocycles

**Divergent synthesis of 1-trifluoromethylated cyclopenta[*b*]indoles:** Cyclopenta[*b*]indole frameworks are prominent structural motifs widely encountered in biologically active natural products and pharmaceutical compounds, where their rigid fused-ring architecture contributes to diverse biological activities [51]. In particular, trifluoromethyl-substituted cyclopenta[*b*]indoles **47** are attractive targets in medicinal chemistry because incorporation of the CF_3_ group can significantly modulate metabolic stability and bioactivity [52].

**Scheme 14:**
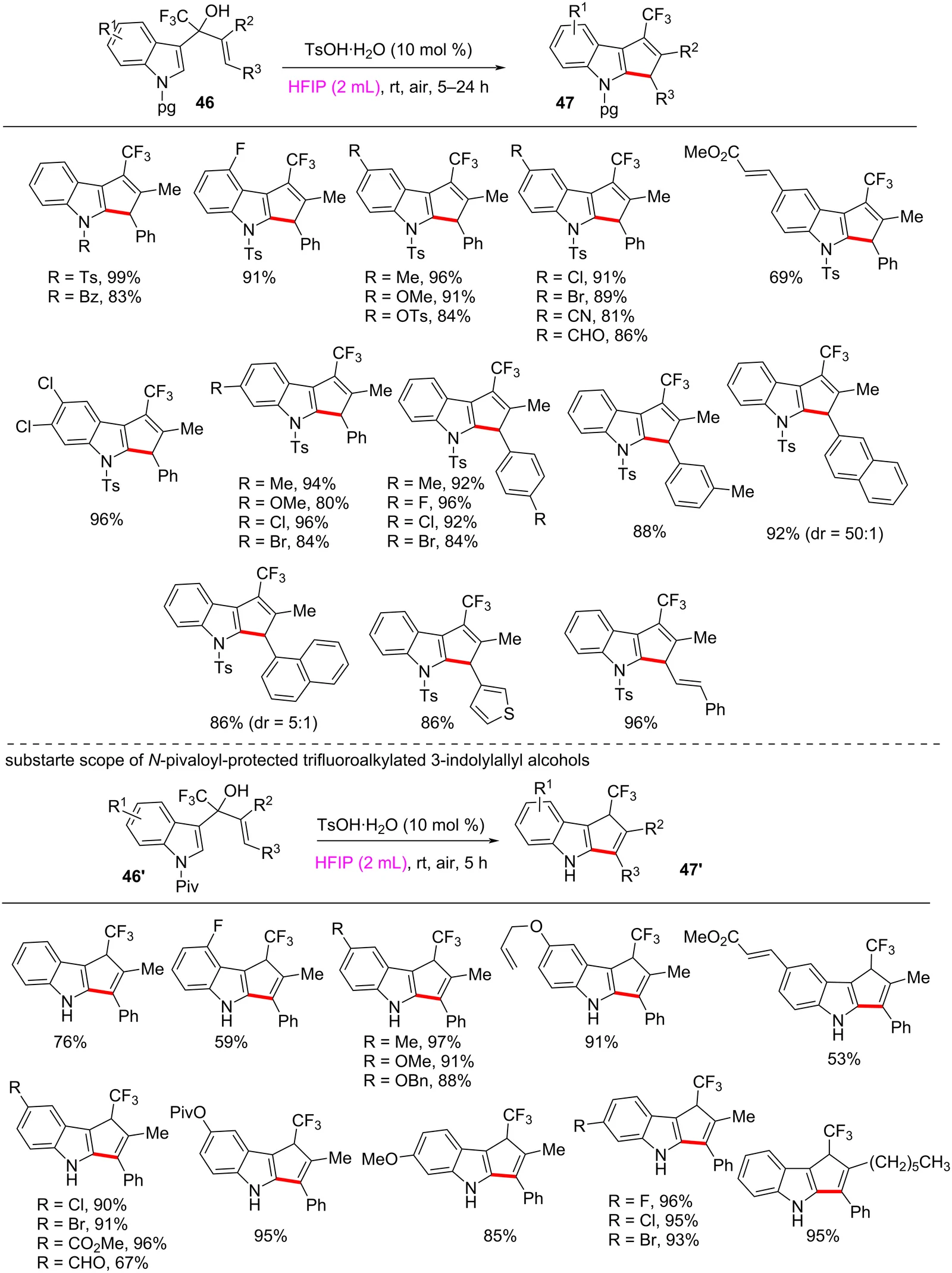
Substrate scope for the synthesis of 1-trifluoromethylated cyclopenta[*b*]indoles **47**.

An efficient Brønsted acid-catalyzed dehydrative Nazarov-type cyclization has been developed by Rao and co-workers for the divergent synthesis of 1-trifluoromethylated cyclopenta[*b*]indoles **47** from CF_3_-substituted 3-indolylallyl alcohols **46** (Scheme 14) [53]. Remarkably, product selectivity is governed by the nature of the indole NH-protecting group. Arylsulfonyl-protected substrates selectively furnish protected cyclopenta[*b*]indoles, whereas pivaloyl-protected analogues undergo further deacylative rearrangement to generate NH-free cyclopenta[*b*]indoles with altered alkene geometry. This tunable chemoselectivity highlights the versatility of the methodology and enables access to two distinct classes of valuable fluorinated scaffolds from common precursors. This strategy offers operational simplicity, scalable synthesis, mild reaction conditions, and controllable product divergence, providing a practical route to structurally diverse trifluoromethylated cyclopenta[*b*]indoles. Wherein, HFIP acts not only as a solvent but also as a key promoter in the reaction, where its combination with TsOH is proposed to generate the highly active catalytic species TsOH(H_2_O)*_m_*(HFIP)*_n_*, thereby enhancing the overall catalytic efficiency of the transformation.

The reaction proceeds under mild metal-free conditions and demonstrates excellent functional-group compatibility. A broad range of substrates bearing electron-donating and electron-withdrawing substituents on the indole core are efficiently transformed into the corresponding cyclopenta[*b*]indoles in generally good to excellent yields (Scheme 14). Sensitive functionalities such as formyl, α,β-unsaturated ester, halogen, cyano, and alkene groups are well tolerated, underscoring the mildness of the conditions. Variations in alkyl substituents and aryl groups on the alkenyl fragment are also accommodated, although sterically demanding naphthyl and heteroaryl substrates can lead to regio- or diastereomeric mixtures. In contrast, less substituted or unactivated allylic alcohols prove unsuitable for the transformation.

**Scheme 15:**
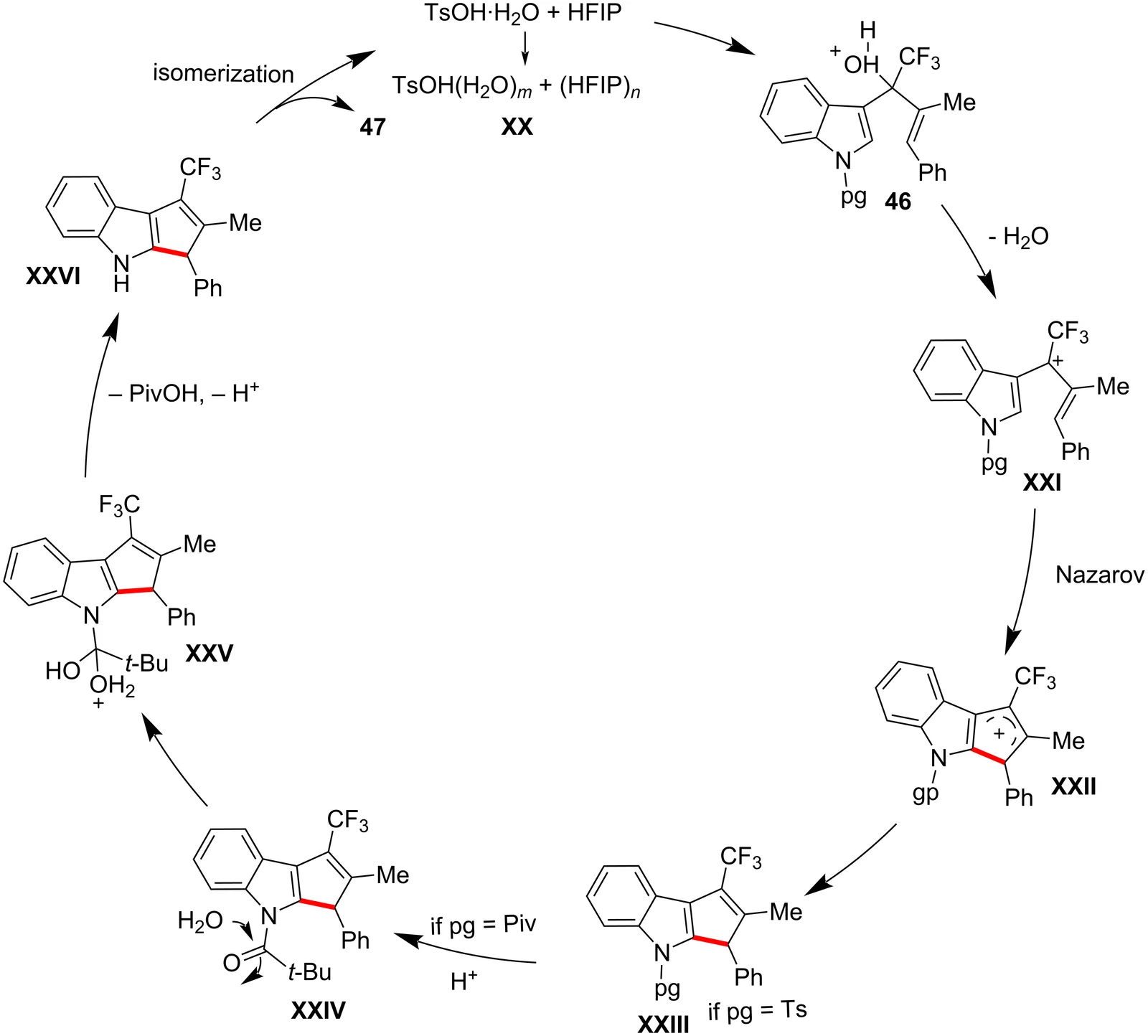
Proposed mechanism for the synthesis of trifluoroalkylated 3-indolylallyl alcohols.

Mechanistically, the reaction involves formation of a highly active TsOH/HFIP catalytic species **XX** that promotes dehydration of the allylic alcohol **46** to generate a carbocation intermediate **XXI** (Scheme 15), followed by Nazarov-type 4π electrocyclization (**XXII**). In the case of pivaloyl-protected substrates, subsequent hydrolysis of **XXIV** and alkene isomerization of **XXVI** lead to NH-free products **47**.

*Synthesis of fluorescent quinoline-fused pyrroles*: Quinolines and pyrroles are among the most important nitrogen-containing heterocycles in medicinal chemistry, and hybrid structures incorporating both motifs frequently display significant biological and photophysical properties [54]. In particular, quinoline-fused pyrroles are present in numerous natural products and synthetic bioactive molecules, motivating the development of efficient and sustainable approaches for their synthesis [55].

**Scheme 16:**
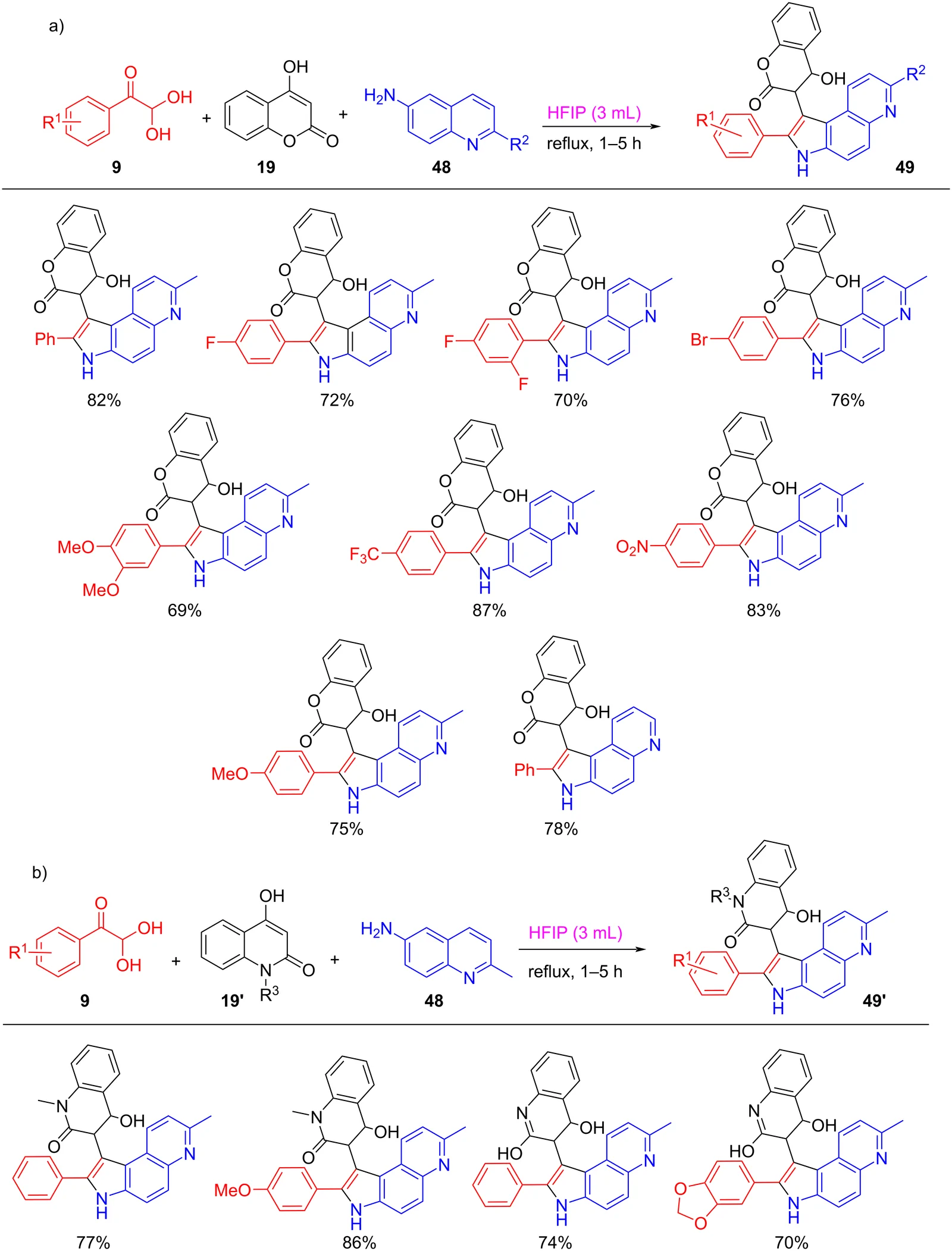
a) Substrate scope for the synthesis of coumarin containing quinoline-fused pyrroles **49**. b) Substrate scope for the synthesis of 4-hydroxy-1-methylquinolone/quinoline-2,4-diol **49’**.

A metal-free multicomponent strategy has been developed by Choudhury and co-workers for the regioselective synthesis of quinoline- and isoquinoline-fused pyrroles **49** using arylglyoxals **9**, cyclic 1,3-dicarbonyl compounds **19**, and aminoquinolines **48** in HFIP under reflux conditions (Scheme 16) [56]. In this transformation, HFIP acts simultaneously as solvent and promoter, facilitating rapid assembly of the fused heterocyclic framework without the need for external catalysts. The protocol combines operational simplicity with straightforward product isolation and provides direct access to structurally complex fused pyrroles containing multiple biologically relevant motifs. The combination of broad substrate compatibility, short reaction times, easy purification, and incorporation of multiple pharmacologically important heterocyclic units underscores the utility of this HFIP-mediated methodology for the synthesis of complex fused N-heterocycles.

The methodology exhibits broad substrate scope and good functional-group tolerance (Scheme 16a). Arylglyoxals bearing both electron-donating and electron-withdrawing substituents participate efficiently, although electron-deficient substrates generally afford higher yields than electron-rich analogues, likely due to enhanced electrophilicity of the glyoxal component. Halogenated, trifluoromethyl-, nitro-, methoxy-, and naphthyl-substituted glyoxals are all compatible with the reaction conditions. Variation of the aminoquinoline and cyclic 1,3-dicarbonyl partners further expands structural diversity, enabling the synthesis of a range of quinoline- and isoquinoline-fused pyrroles in moderate to very good yields (Scheme 16b).

**Scheme 17:**
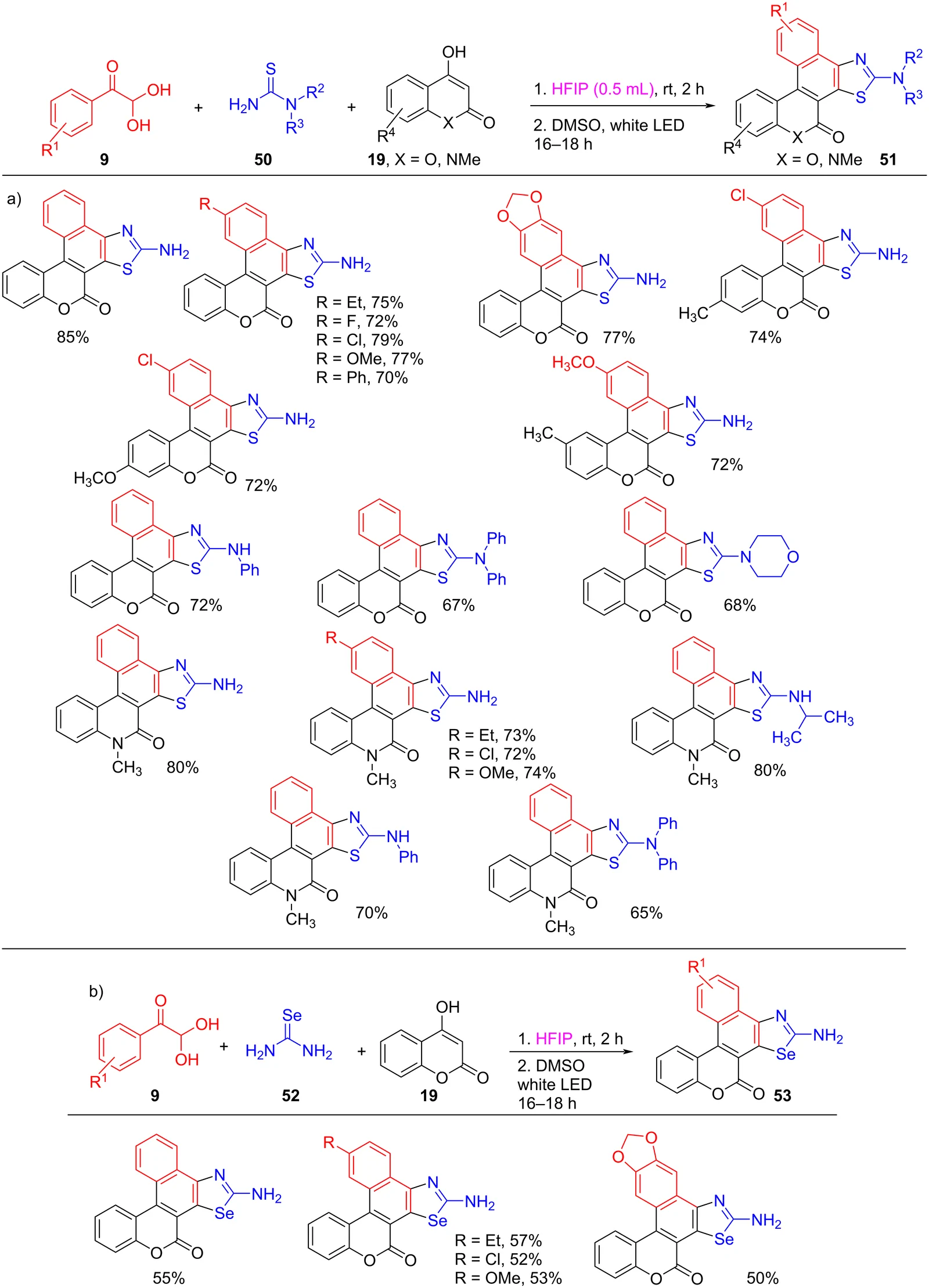
a) Substrate scope for the synthesis of polycyclic-fused aminothiazoles **51**. b) Substrate scope for the synthesis of polycyclic aromatic aminoselenazoles **53**.

**Synthesis of polycyclic-fused aminothiazoles and amino-selenazoles:** The development of fused aminothiazole and aminoselenazole scaffolds through multicomponent reactions (MCRs) has emerged as an efficient strategy for accessing structurally complex and biologically relevant heterocycles [57]. In this context, a visible-light-assisted, HFIP-mediated one-pot protocol was reported by Choudhury and co-workers for the synthesis of pentacyclic-fused aminothiazoles **51** and aminoselenazoles **53** from arylglyoxals **9**, thioureas/selenoureas **50/52**, and 4-hydroxycoumarin or 4-hydroxy-1-methyl-2-quinolone derivatives (Scheme 17) [22]. The significant enhancement in reaction efficiency highlights the distinctive role of HFIP in promoting the transformation, which can likely be attributed to its strong hydrogen-bond-donating ability and high polarity. The methodology combines an initial MCR cyclization with subsequent photocyclization under white LED irradiation, enabling rapid construction of multiple C–C, C–N, and C–S/Se bonds under mild and metal-free conditions. This strategy offers improved atom economy, operational simplicity, and the use of visible light as a sustainable energy source. Critically, the protocol demonstrates several advantages over traditional transition-metal-catalyzed annulation approaches, including avoidance of metal contaminants, ambient reaction conditions, and minimal waste generation, with water as the only benign byproduct. However, the reduced efficiency observed for selenourea substrates and sterically hindered thioureas indicates certain limitations in substrate compatibility.

A broad substrate scope was demonstrated, with both electron-donating and electron-withdrawing substituents on arylglyoxals providing the desired fused aminothiazoles in generally good to excellent yields (Scheme 17a). Thiourea derivatives bearing mono-substitution showed higher efficiency, whereas *N,N*-disubstituted thioureas resulted in comparatively diminished yields, likely due to steric and electronic effects. Similarly, coumarin-based substrates consistently outperformed 4-hydroxy-1-methyl-2-quinolone analogues, which furnished slightly lower yields under identical conditions. Notably, replacement of thiourea with selenourea successfully extended the methodology to aminoselenazole synthesis, although these reactions afforded only moderate yields, highlighting the comparatively lower reactivity and stability of selenium-containing intermediates (Scheme 17b).

#### Spiro heterocyclic rings

**Scheme 18:**
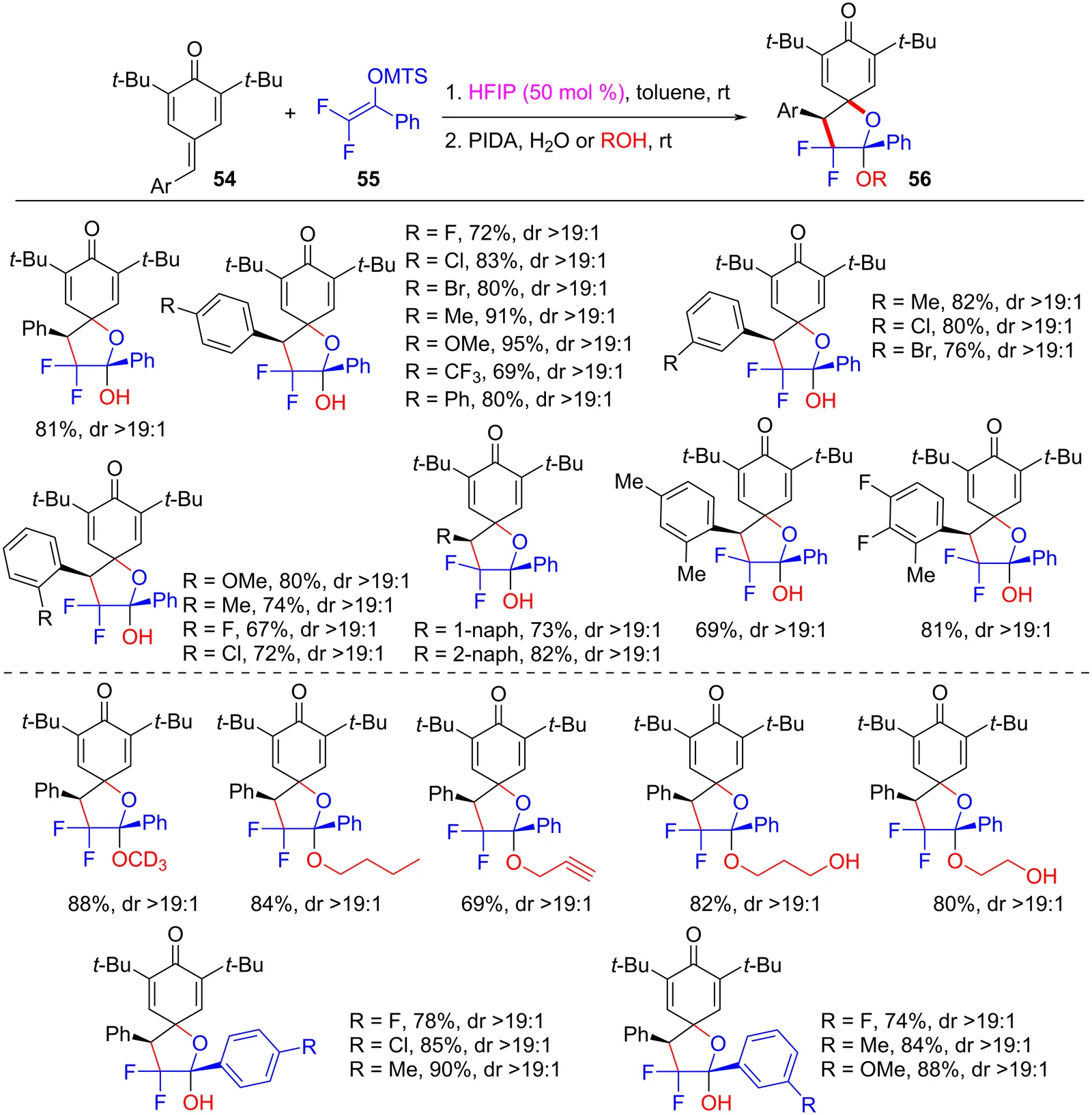
Substrate scope for the synthesis of spiro[4.5]cyclohexadienones **56**.

**Construstion of five-six membered spiro heterocyclic rings: ***Synthesis of fluorine-containing multisubstituted oxaspiro[4,5]cyclohexadienones: para*-quinone methides (*p*-QMs) are highly reactive intermediates widely encountered in natural products and bioactive systems [58], and they serve as versatile synthons in modern organic synthesis due to their ability to participate in diverse bond-forming processes [59]. In this context, a one-pot sequential cascade strategy has been developed by Wang and co-workers for the construction of fluorinated multisubstituted oxaspiro[4,5]cyclohexadienones **56** using *p*-QMs **54** and difluoroenoxysilanes **55** under HFIP catalysis, followed by oxidative dearomatization (Scheme 18) [60]. Mechanistically, the reaction proceeds through an HFIP-promoted 1,6-conjugate addition of difluoroenoxysilanes to *p*-QMs **54**, followed by intramolecular hemiacetalization and oxidative dearomatization using PhI(OAc)_2_, delivering the spirocyclic framework with high diastereoselectivity. In contrast to conventional solvent roles, HFIP functions as a mild Brønsted acid catalyst, enabling efficient cascade reactivity under relatively mild conditions and highlighting the emerging catalytic potential of fluorinated alcohols in complex molecule construction. This work offers superior atom economy, operational simplicity, and rapid access to fluorine-rich spirocyclic architectures that are of significant interest in medicinal chemistry and drug discovery.

The methodology exhibits a broad substrate scope (Scheme 18). A variety of *p*-QMs bearing electron-donating, electron-withdrawing, and neutral substituents participate effectively, generally affording moderate to good yields, although electron-deficient systems show slightly reduced efficiency. Sterically demanding naphthyl-derived substrates are also compatible, while disubstituted and heterocyclic *p*-QMs, although not shown in Scheme 18, further expands the reported structural diversity. In parallel, difluoroenoxysilanes with varied electronic and steric profiles are well tolerated, enabling access to a wide range of fluorinated spirocyclic products. Additionally, diverse alcohol nucleophiles, including deuterated methanol and diols, participate successfully, whereas amines and thiols are unreactive under the oxidative conditions.

**Scheme 19:**
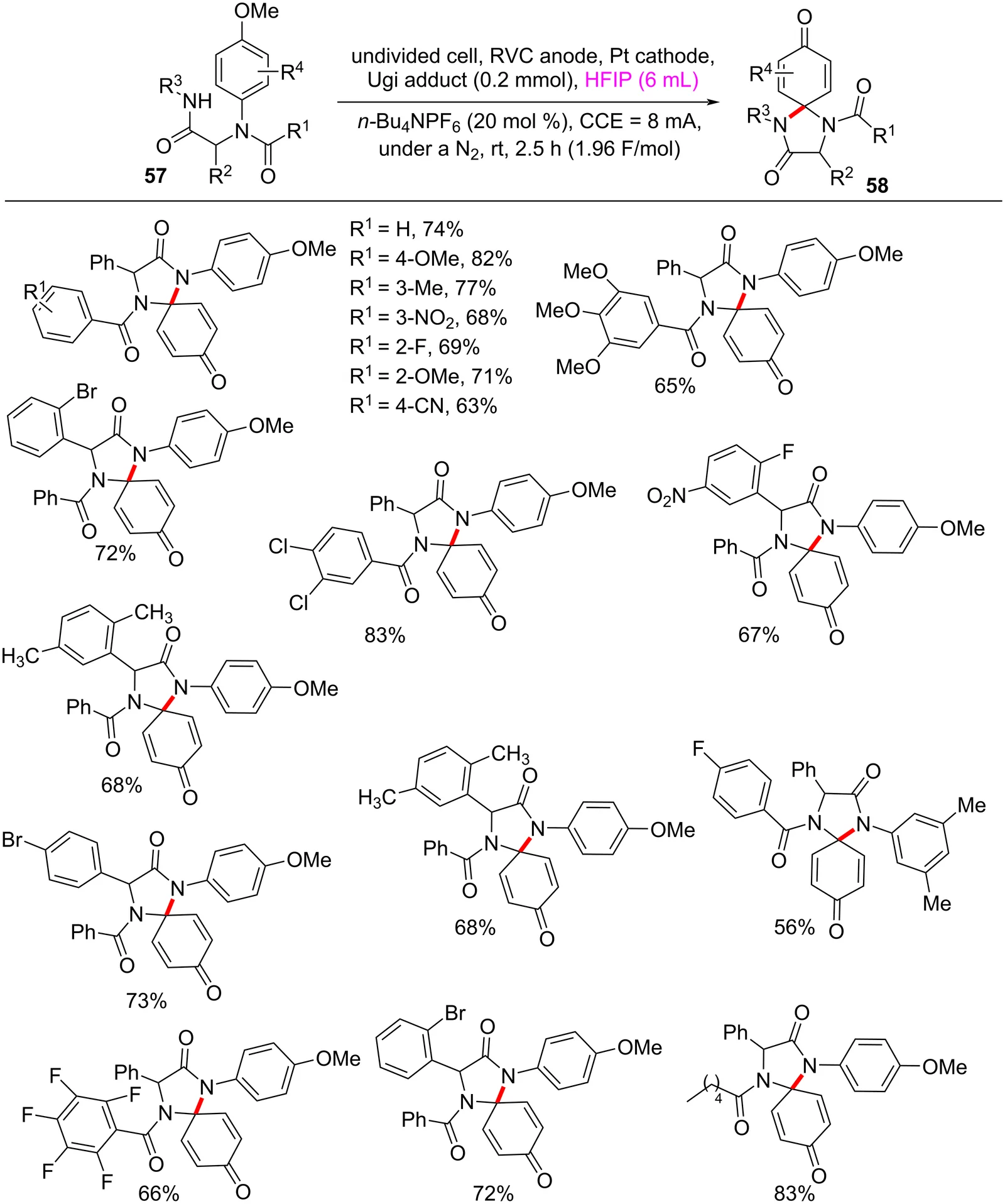
Substrate scope for synthesis of diverse 4-imidazolidinones **58**.

*Synthesis of 4-imidazolidinones via electrochemical and post-Ugi cyclization method:* Organic electrosynthesis has emerged as a sustainable and powerful strategy for generating reactive radical intermediates under mild conditions [61], enabling efficient construction of structurally complex molecules without the need for stoichiometric oxidants or metal catalysts. In this context, an electrochemical approach has been developed by Sharma and co-workers for the synthesis of spirocyclic 4-imidazolidinones **58** through post-Ugi cyclization of bis-amide intermediates via N-centered radical-mediated intramolecular *ipso* cyclization (Scheme 19) [62].

The transformation demonstrates a broad substrate scope and notable functional-group tolerance (Scheme 19). Aromatic substrates bearing electron-donating and electron-withdrawing substituents at various positions react efficiently, while electrochemically sensitive groups such as bromo, nitro, cyano, methoxy, and alkenyl functionalities remain intact. Both aromatic and aliphatic carboxylic acid-derived Ugi adducts participate successfully, and structurally diverse aldehydes and isocyanides further expand the accessible spirocyclic library. In contrast, phenyl and aliphatic isocyanide-derived substrates proved ineffective, likely due to insufficient stability of the intermediate amidyl radicals.

**Scheme 20:**
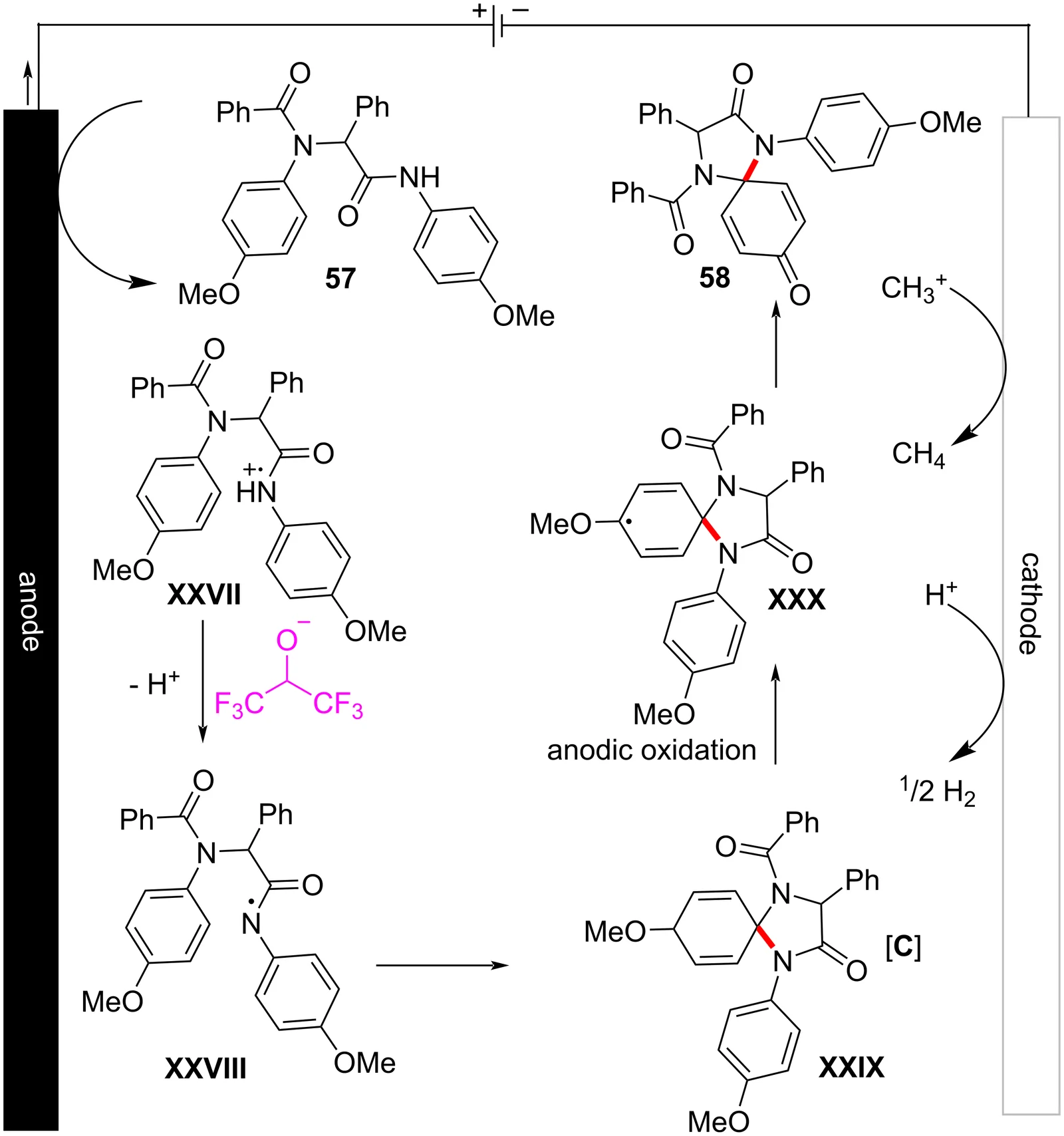
Proposed mechanism for the synthesis of diverse 4-imidazolidinones **58**.

The method employs constant-current electrolysis in an undivided carbon-platinum cell to generate amidyl radicals directly from Ugi adducts. Mechanistically, anodic oxidation produces an N-centered radical **XXVIII** that undergoes intramolecular cyclization (**XXIX**) and subsequent oxidation (**XXX**) to furnish the spirocyclic scaffold **58** following demethylation. HFIP plays a dual role of facilitating deprotonation and stabilizing reactive intermediates, while its recyclability further enhances the sustainability of the protocol (Scheme 20). Compared with traditional oxidative dearomatization methods, this electrochemical strategy avoids external catalysts, oxidants, and additives, broad functional-group tolerance, scalability, and efficient access to structurally diverse N-heterocycles, highlighting the growing utility of electro-organic synthesis in heterocyclic chemistry and late-stage functionalization.

*Synthesis of highly functionalized spirolactams:* Spirocyclic compounds are prominent structural motifs in natural products, pharmaceuticals, and functional materials due to their rigid three-dimensional architectures and unique biological properties [63]. Among them, spiro[4.5]trienones have attracted considerable attention as versatile intermediates for the construction of complex molecular frameworks [64]. In this regard, the merger of multicomponent Ugi chemistry with electro-organic synthesis has provided an efficient platform for the rapid assembly of structurally diverse azaspirocyclic systems.

**Scheme 21:**
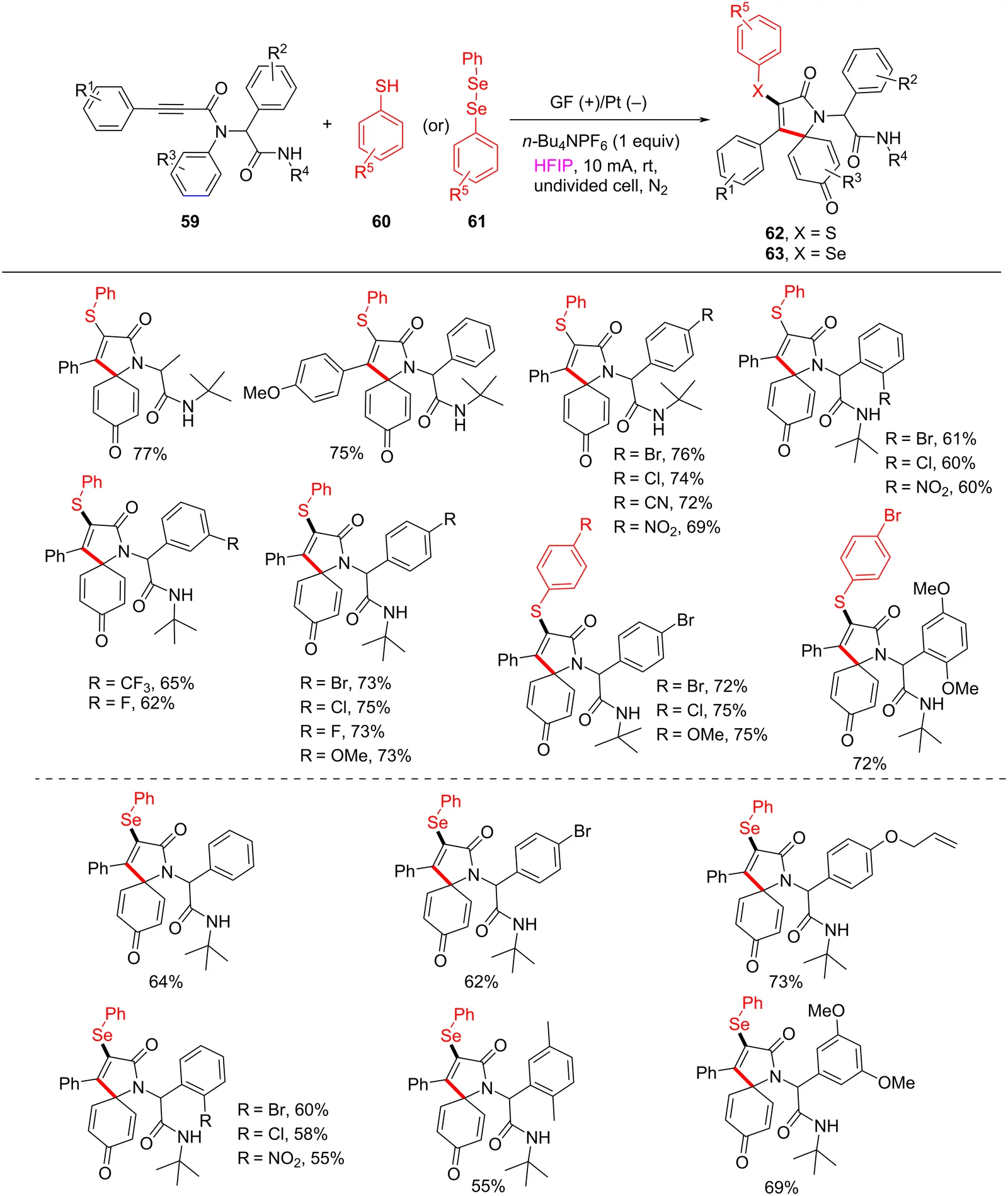
Substrate scope of electrochemical spirocyclization and selinidation conversions.

A catalyst- and metal-free electrochemical strategy has been developed by Sharma and co-workers for the synthesis of C–S, C–Se, and C–C=O functionalized spirolactams from Ugi-derived bisamides through oxidative dearomatizing spirocyclization [65]. The transformation proceeds under mild electrochemical conditions via a radical-mediated cascade cyclization, enabling the formation of azaspiro-fused bicyclic scaffolds **62** and **63** (Scheme 21). The detailed regioisomeric and diastereomeric substrate scope is not shown in Scheme 21; readers are referred to the original report for further details. This electrochemical method offers a cleaner and more sustainable alternative by avoiding external oxidants, catalysts, and metals. The successful application of a micro-electro-flow reactor for gram-scale synthesis further demonstrates the practicality and scalability of the protocol.

The protocol exhibits a broad substrate scope and excellent functional-group tolerance (Scheme 21). Ugi adducts derived from various acids, aldehydes, amines, and isocyanides undergo smooth electrochemical cyclization irrespective of the electronic nature of aromatic substituents. Both electron-rich and electron-deficient aldehydes are well tolerated, while substituted anilines and diverse isocyanides further expand structural diversity. In addition, a wide range of thiols and diselenides bearing halogen, methoxy, and naphthyl substituents participate effectively, affording sulfur- and selenium-containing azaspirocycles in moderate to excellent yields. The methodology also accommodates aliphatic isocyanide-derived substrates and enables further transformation of the spirocyclic products into fully fused tricyclic systems.

**Scheme 22:**
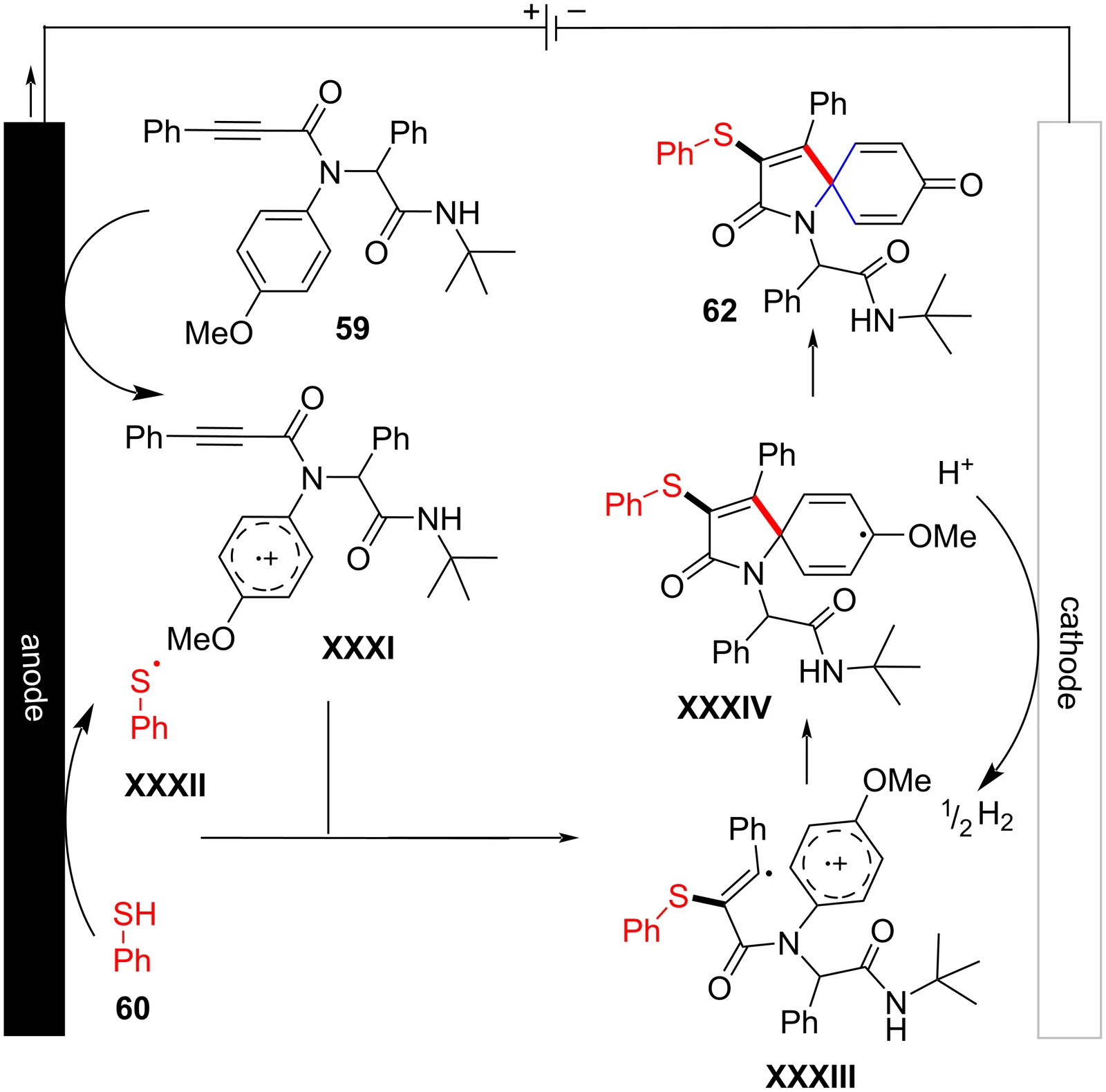
Proposed mechanism for electrochemical spirocyclization conversions.

Mechanistic investigations suggest that anodic oxidation simultaneously generates radical intermediates (**XXXI** and **XXXII**) from both the Ugi adduct and thiol/diselenide coupling partners, followed by radical addition to give **XXXIII**, cyclization, and demethylation of **XXXIV** to furnish the final spirocyclic products **62** and/or **63** (Scheme 22).

**Scheme 23:**
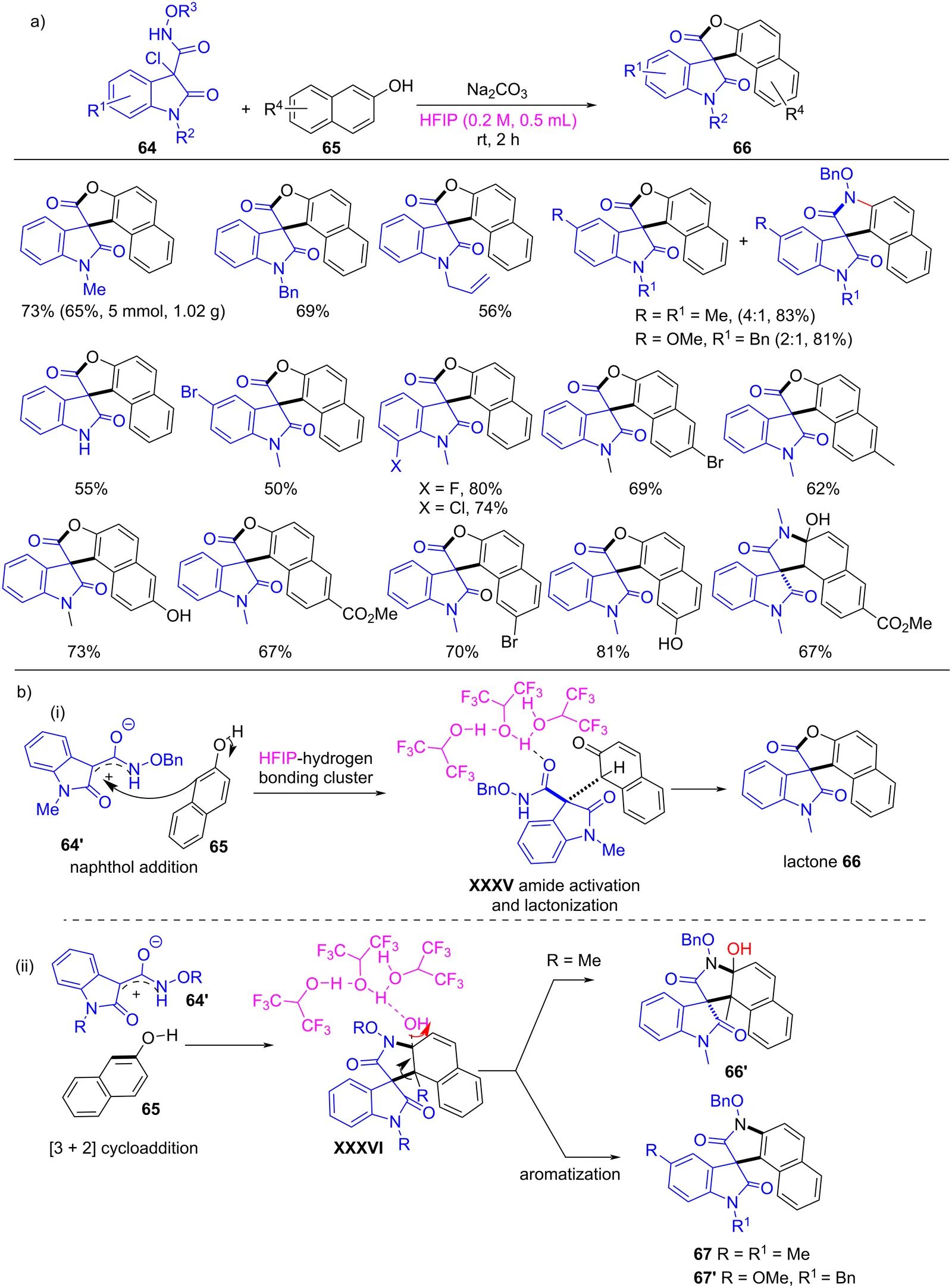
a) Substrate scope for various oxindolyl α-halohydroxamate **64** and 2-naphthols **65.** b) Proposed mechanism for lactonization vs [3 + 2] cycloaddition–aromatization sequence.

**Five-five spiro-fused heterocyclic frameworks: ***Synthesis of spirooxindole-γ-lactones:* Among spirocyclic frameworks, spirooxindoles constitute an especially important class owing to their broad biological activities and frequent occurrence in natural products and bioactive molecules [66]. In particular, spirooxindole-γ-lactones **66** and bis-spirooxindole **67**, in which the oxindole core is connected through the C3 position to an oxygen-containing five-membered ring, have emerged as attractive synthetic targets (Scheme 23a).

An efficient HFIP-mediated strategy has been developed by Saha and co-workers for the synthesis of spirooxindole-γ-lactones **66** through the reaction of transient oxindolyl azaoxyallyl **64** cations with β-naphthols **65** [67]. In this transformation, HFIP acts not only as the sole activator for generating the azaoxyallyl cation but also facilitates the key lactonization step through hydrogen-bond-assisted activation.

The methodology demonstrates a broad substrate compatibility. Oxindoles bearing various N-substituents, including free NH derivatives, undergo smooth annulation, while substitutions at different positions of the oxindole ring significantly influence product distribution. Electron-rich C5-substituted oxindoles partially favor competing bis-spirooxindole formation, whereas halogen substitution, particularly at the C7 position, improves reaction efficiency and selectivity toward lactones. A variety of substituted β-naphthols containing electron-donating, electron-withdrawing, and halogen substituents are also well tolerated, generally affording moderate to good yields. Interestingly, sterically modified naphthol substrates can alter the reaction pathway, leading exclusively to dearomative [3 + 2] cycloaddition products (Scheme 23a). Overall, this HFIP-enabled strategy provides a versatile and selective route to biologically relevant spirooxindole-γ-lactones and related fused oxygen heterocycles, while also expanding the synthetic utility of azaoxyallyl cation intermediates in complex spirocycle synthesis.

Mechanistically, nucleophilic addition of β-naphthol **65** to the azaoxyallyl intermediate **64’** is followed by rapid intramolecular cyclization (**XXXV**) to furnish the spiro-lactone scaffold **66**. In contrast to competing [3 + 2] cycloaddition pathways that generate bis-spirooxindole products **67**, lactonization predominates in most cases, highlighting the high chemoselectivity of the protocol (Scheme 23b).

**Five-six spiro-fused heterocyclic frameworks: ***PIDA-Promoted/HFIP-controlled dearomative spirocyclization of phenolic ketones:* Spirocyclohexadienones are important structural motifs found in numerous biologically active natural products and pharmaceutical compounds [68], and they also serve as versatile intermediates for the synthesis of architecturally complex molecules. Dearomative spirocyclization has emerged as a powerful strategy for constructing these highly functionalized three-dimensional frameworks from simple aromatic precursors [69].

**Scheme 24:**
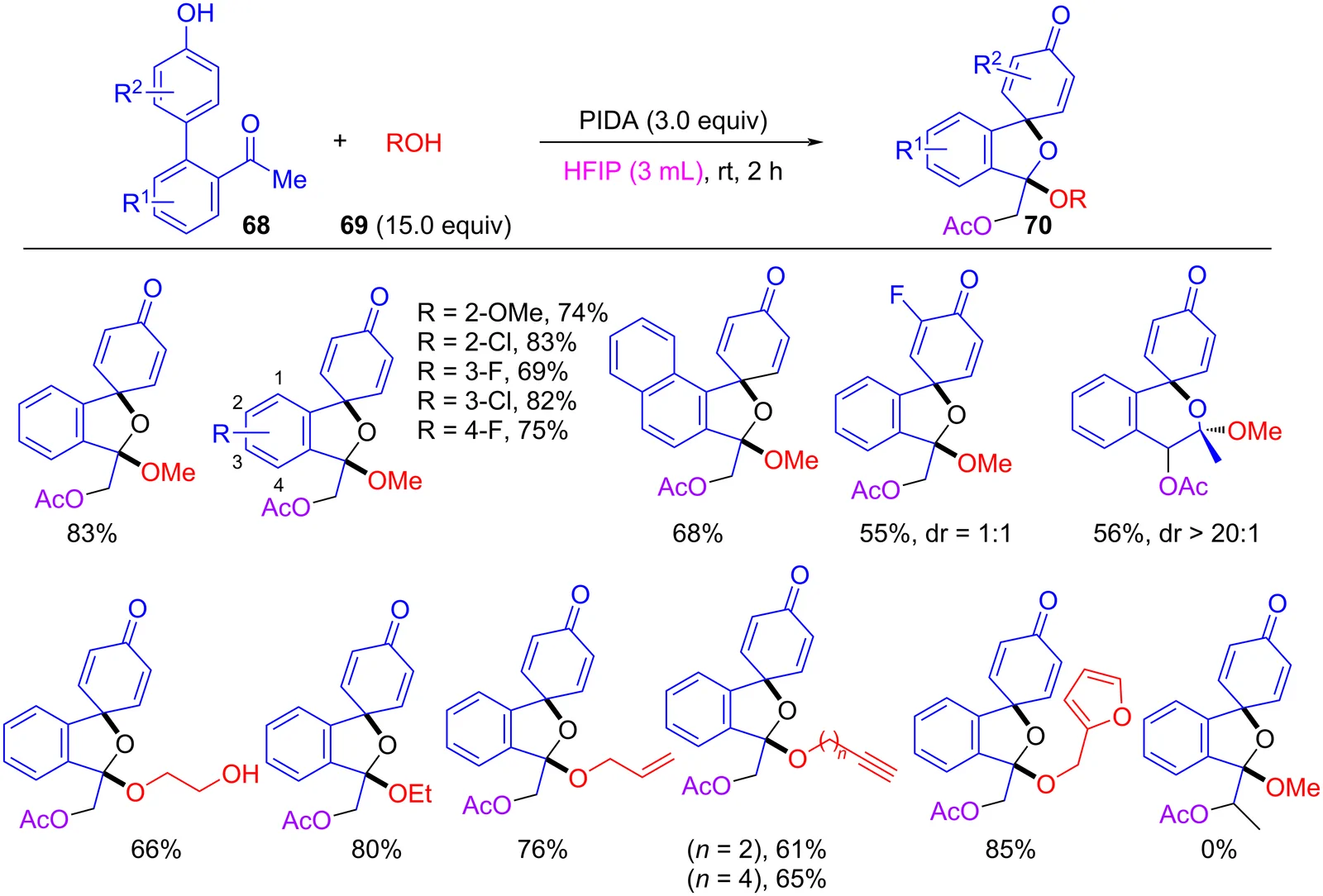
Scope of substrates for the synthesis of products **70**.

In this context, a PIDA (phenyliodine(III) diacetate)-promoted and HFIP-controlled dearomative spirocyclization of phenolic ketones **68** has been developed by Peng and co-workers [70], enabling the efficient synthesis of spirocyclohexadienonic ketals **70** and their acetoxylated analogues under mild conditions (Scheme 24). The combined use of alcohols **69** as the nucleophile and HFIP as the solvent proved crucial for controlling the cascade process, highlighting the distinctive role of HFIP in stabilizing cationic intermediates and promoting selective dearomatization.

The methodology exhibits good substrate generality, with a variety of substituted phenolic ketones and functionalized alcohols participating effectively to afford the desired products in moderate to excellent yields under mild oxidative conditions (Scheme 24). Both electronic and steric variations on the aromatic framework are well tolerated, and the process provides good regio- and diastereoselectivity in selected cases. However, deviations in substitution patterns, particularly at specific alkyl substituents, negatively influence reactivity, indicating some substrate limitations. Mechanistic studies suggest that the transformation proceeds through the generation of a spirocyclohexadienone oxocarbenium intermediate, which undergoes nucleophilic trapping to furnish the final spirocyclic products. In situ-generated hypervalent iodine catalytic version is observed in this phenomenon.

#### Saturated and non-aromatic heterocyclic rings

**Scheme 25:**
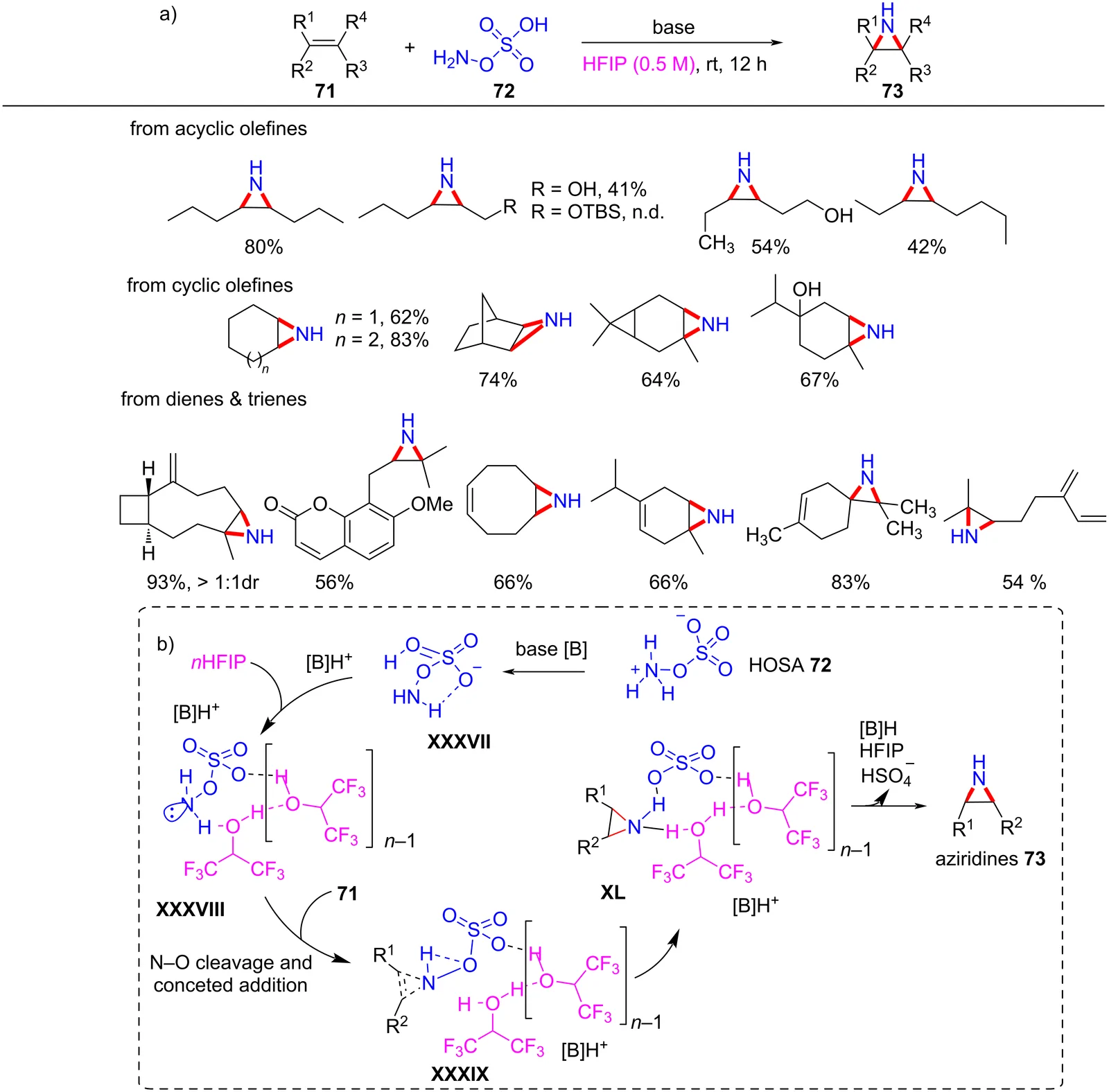
a) Functional group compatibility for the products **73**. b) Proposed mechanism.

**Three-membered saturated heterocycle construction: ***Synthesis of N–H aziridines from unactivated olefins:* Aziridines, the smallest nitrogen-containing heterocycles, are important privileged scaffolds frequently encountered in natural products and biologically active molecules, including antitumor and antimicrobial agents [71]. A practical and efficient intermolecular aziridination protocol has been developed by Wang and co-workers using hydroxylamine-*O*-sulfonic acid (HOSA, **72**) as the aminating reagent in an HFIP-based solvent system with pyridine or piperidine as base additives (Scheme 25) [72]. The method enables direct conversion of a broad range of alkenes **71** into free N–H aziridines **73** under mild and operationally simple conditions. This HFIP-enabled protocol offers a simple, scalable, and metal-free alternative for accessing free N–H aziridines directly from unactivated alkenes. The combination of mild conditions, broad alkene scope, high regioselectivity, and gram-scale applicability underscores its potential utility for synthetic and medicinal chemistry applications.

The protocol exhibits broad substrate scope and particularly high efficiency with nonactivated alkenes (Scheme 25a). Mono-, di-, tri-, and tetrasubstituted alkenes all participate effectively, generally affording moderate to excellent yields. Electron-rich alkenes display enhanced reactivity, while terminal olefins are comparatively less reactive (not shown in the Scheme 25a). Cyclic and bridged cycloalkenes are well tolerated and often provide products with high stereoselectivity. The methodology also demonstrates remarkable regioselectivity in polyunsaturated systems, where aziridination selectively occurs at the more electron-rich double bond, even in the presence of multiple olefinic sites. Importantly, dienes and trienes undergo exclusive monoaziridination despite excess aminating reagent, highlighting the chemoselective nature of the process. Functional group compatibility is generally good, although free hydroxy groups can alter reactivity or lead to competing intramolecular ring-opening processes. In some substrates, protecting groups or changes in base were necessary to achieve optimal outcomes. Particularly noteworthy is the observation of cascade aziridination/ring-opening sequences in allylic alcohol substrates, illustrating the synthetic versatility of the generated aziridines.

Mechanistically, the transformation is proposed to proceed through base-assisted activation of HOSA (**72**) followed by formation of a hydrogen-bonded HFIP cluster **XXXVIII** that facilitates N–O bond cleavage to generate a highly electrophilic aminating species **XXXIX**. Subsequent concerted addition (**XXXIX**) across the alkenes **71** furnish aziridine products **73**. The critical role of HFIP appears to arise from its ability to stabilize reactive intermediates through extensive hydrogen-bonding interactions, thereby promoting efficient aziridination under metal-free conditions (Scheme 25b).

**Scheme 26:**
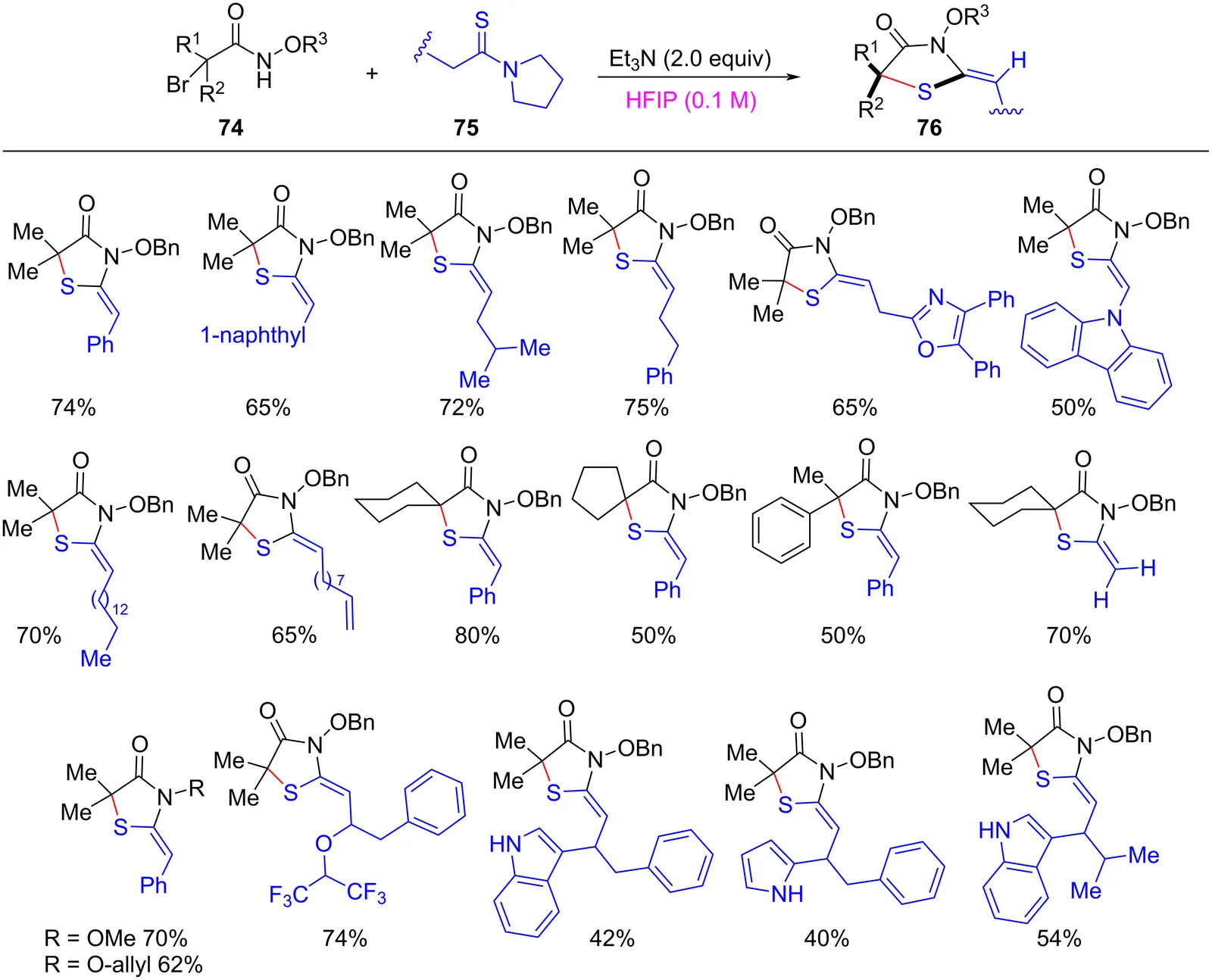
Substrate scope for the synthesis of alkylidene-4-thiazolidinone **76**.

**Five-membered saturated heterocycle construction:***Synthesis of structurally diverse alkylidene-4-thiazolidinones/selenazolidinones:* Thiazolidine-4-ones are an important class of S,N-heterocycles widely associated with diverse biological activities, including antimicrobial, antiviral, anticancer, and anti-inflammatory properties [73]. A mild and efficient HFIP-promoted one-pot strategy has been developed by Saha and co-workers for the synthesis of structurally diverse alkylidene-4-thiazolidinones **76** through cycloaddition of α-bromohydroxamates **74** with thioamides **75** (Scheme 26) [74], followed by spontaneous E_1_-type elimination of the C2 amino group from the intermediate cycloadduct. Notably, the transformation proceeds under metal- and acid-free conditions without the need for amine quaternization, while enabling stereoselective formation of the exocyclic olefin.

This protocol offers a concise, modular, and operationally simple alternative for accessing highly functionalized alkylidene-4-thiazolidinones **76** and related heterocycles. The combination of broad substrate compatibility, stereoselective olefin formation, scalability, and extension to spirocyclic and selenium-containing systems underscores the versatility and synthetic value of the methodology.

The methodology displays a broad substrate scope and excellent functional-group tolerance (Scheme 26). A variety of aryl, naphthyl, and alkyl-substituted thioamides participate efficiently, including substrates containing terminal or internal alkenes that remain chemoselectively untouched despite the potential for competing cycloaddition reactions. The protocol also accommodates heteroatom-containing side chains, conjugated systems, and drug-derived thioamides, illustrating its applicability for late-stage diversification. Particularly noteworthy is the compatibility of β-hydroxy thioamides, which enables access to conjugated α,β-unsaturated thiazolidinones after oxidation, providing motifs commonly encountered in biologically active molecules [74].

The scope further extends to diverse α-halohydroxamates bearing cycloalkyl, aryl, allyl, *tert*-butyl, and propargyl substituents (not shown in the Scheme 26), while oxindole-derived precursors enable direct synthesis of spirooxindolyl thiazolidinones with medicinally relevant architectures. In addition, the methodology was successfully adapted to the synthesis of selenazolidinones from selenomides, significantly broadening the heterocyclic diversity accessible through this platform. Several downstream transformations, including sulfur oxidation and N–O bond cleavage, further demonstrate the synthetic utility of the products.

Mechanistically, the reaction involves in situ generation of an azaoxyallyl cation that undergoes annulation with thioamides to form aminothiazolidinone intermediates. HFIP plays a central role throughout the process by facilitating azaoxyallyl cation formation, stabilizing carbocationic intermediates, and promoting stereoselective α-deprotonation to furnish predominantly *Z*-configured alkylidene products. The study highlights the multifaceted influence of HFIP in controlling both reactivity and selectivity.

**Scheme 27:**
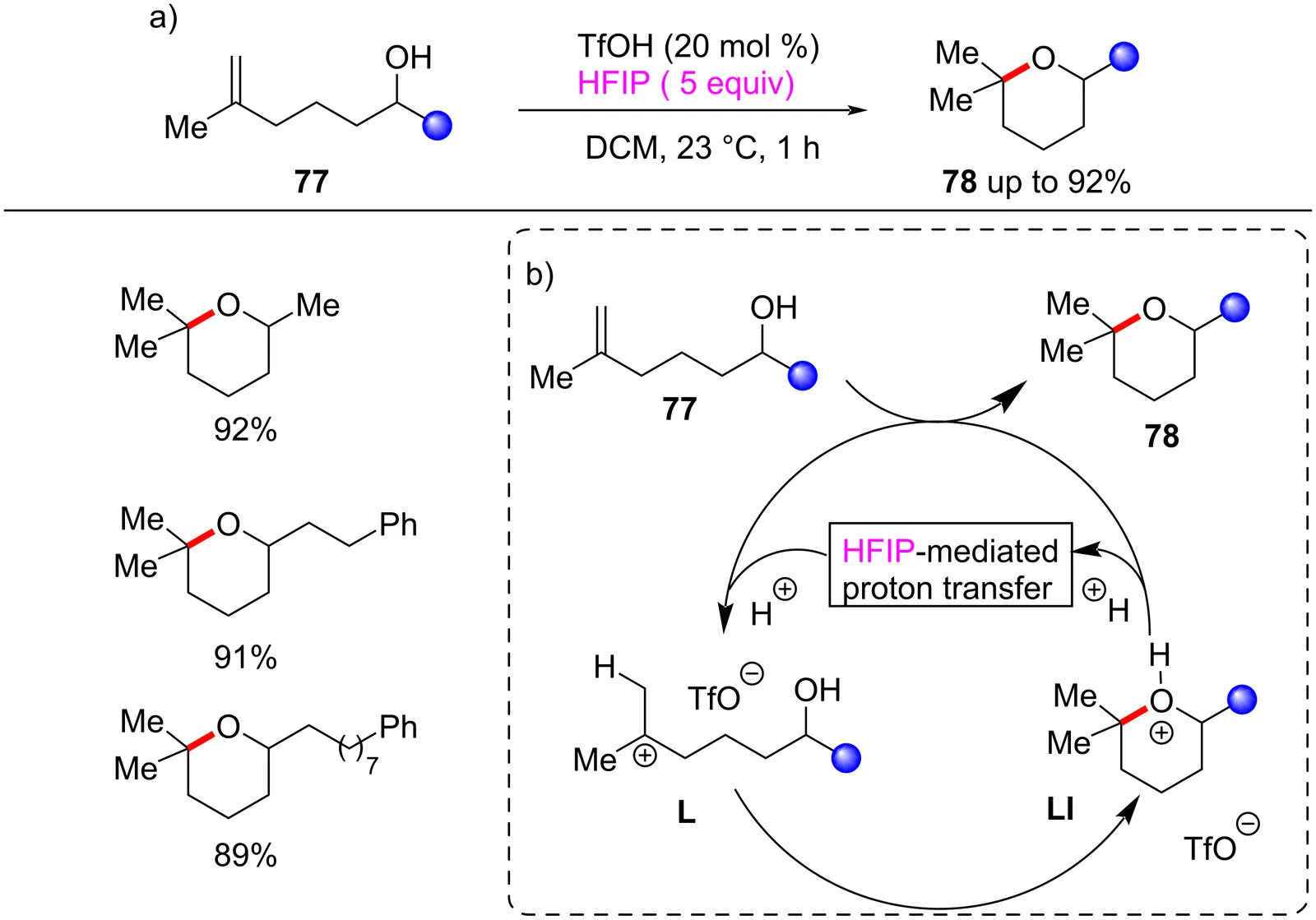
a) Scope of TfOH-catalyzed reaction. b) Proposed mechanism for products **78**.

**Six-membered saturated heterocycle construction:***Synthesis tetrahydropyran derivatives:* Tetrahydropyrans (THPs) and tetrahydrofurans (THFs) are important oxygen-containing heterocycles frequently encountered in natural products, pharmaceuticals, fragrances, and flavoring agents [75]. A mild Brønsted acid-catalyzed intramolecular hydroalkoxylation strategy has been developed by Maulide and co-workers for the regioselective synthesis of trisubstituted tetrahydropyran derivatives **78** using catalytic triflic acid in the presence of HFIP (Scheme 27a) [76].

This approach provides a straightforward and operationally simple route to cyclic ethers with high efficiency and minimal purification requirements. The combination of catalytic Brønsted acid activation and HFIP-mediated proton transfer offers an effective strategy for constructing structurally diverse THP and THF scaffolds under mild conditions.

The methodology exhibits good substrate generality and tolerates both aromatic and aliphatic side chains without significant loss in efficiency (Scheme 27a), affording the desired tetrahydropyrans **78** in consistently high yields. In addition to six-membered ring formation, the protocol also enables access to bridged bicyclic tetrahydrofuran derivatives when dictated by conformational or ring-strain preferences. In one notable case, cyclization selectively produced a THF rather than the corresponding THP, likely due to the unfavorable conformational requirements associated with the alternative six-membered ring system. This observation highlights the influence of substrate geometry and ring stability on regioselectivity.

Mechanistic observations suggest that HFIP plays a crucial role in facilitating proton-transfer processes and stabilizing reactive cationic intermediates, thereby enabling efficient cyclization under catalytic acid conditions and relatively mild reaction parameters (Scheme 27b). The transformation proceeds through protonation of the alkene to generate a tertiary carbocation intermediate **L**, followed by intramolecular nucleophilic attack of the hydroxy group and subsequent deprotonation of **LI** to furnish the cyclic ether product **78**.

**Scheme 28:**
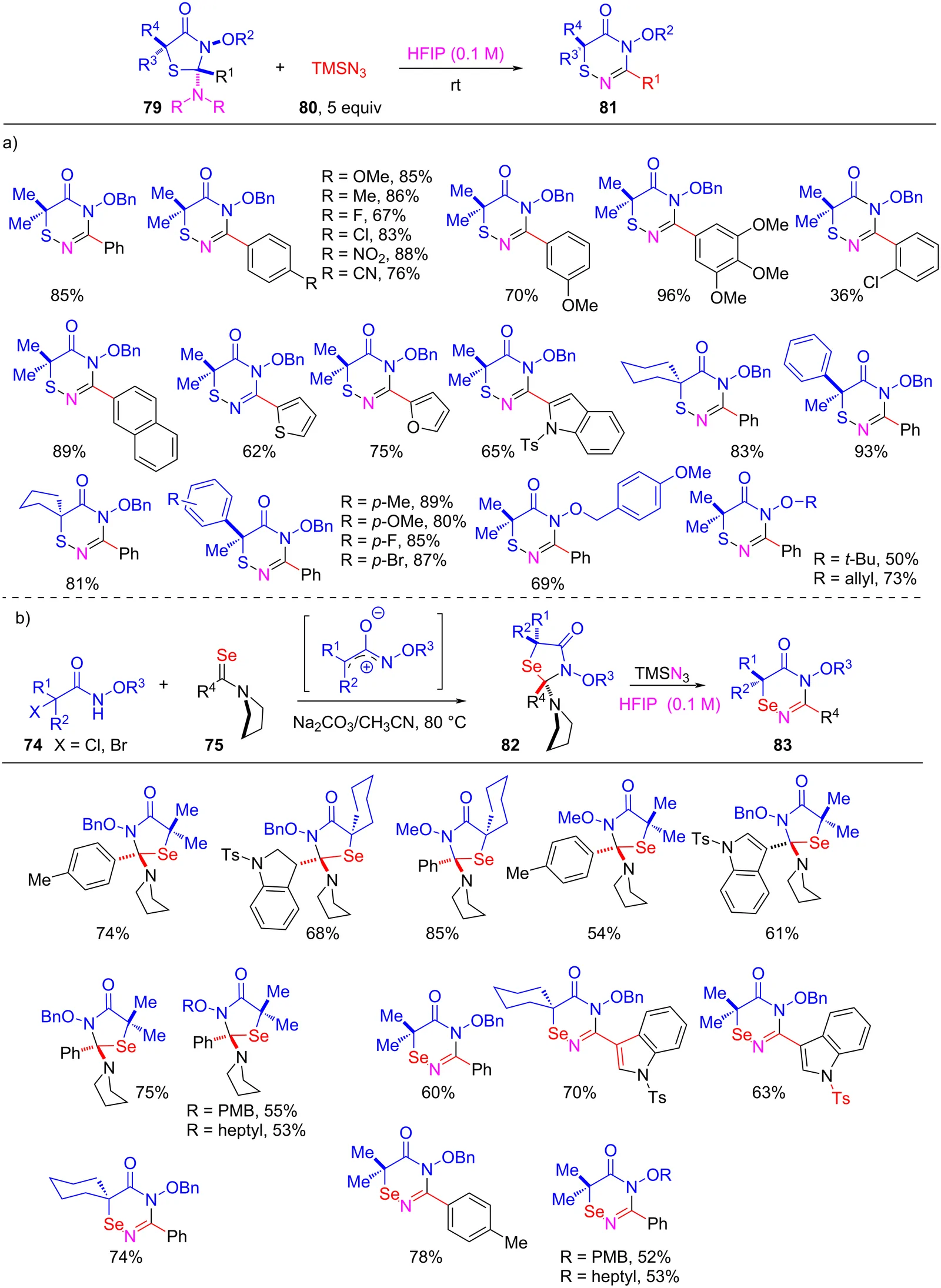
Substrate scope for six-membered (a) S,N-heterocycles **81** and (b) Se,N-heterocycles **83**.

**Mixed five-/six-membered heterocycle construction:***Leveraging the domino skeletal expansion of thia-/selenazolidinones:* Functionalization and skeletal editing of heterocycles remain central strategies in modern organic synthesis and medicinal chemistry [77]. In this context, a highly regioselective domino skeletal-expansion protocol has been developed by Saha and co-workers for the conversion of 2-aminothiazolidinones **79** into six-membered S,N-heterocycles **81** using TMS-azide (**80**) in HFIP at ambient temperature and under metal-free conditions (Scheme 28) [78]. The transformation exploits the latent nucleofugal reactivity of the C2 tertiary amine in thiazolidinones, enabling relay substitution with azide followed by ring expansion through an intermolecular nitrogen-atom transfer process. The mild reaction conditions, broad substrate compatibility, scalability, and applicability to late-stage modification make the methodology particularly attractive for medicinal chemistry and heterocyclic scaffold diversification.

The methodology exhibits a broad substrate scope and tolerates a wide range of substituents on the thiazolidinone framework (Scheme 28a). Electron-donating and electron-withdrawing aryl groups at the C2 position are well accommodated, generally providing the expanded heterocycles in good to excellent yields. Extended π-systems and heteroaryl substituents, including naphthyl, furyl, thienyl, and protected indolyl motifs, are also compatible. In contrast, *ortho*-substituted substrates display diminished efficiency due to steric effects, while free indole NH groups inhibit the transformation. The practicality of the protocol is further demonstrated through gram-scale synthesis and late-stage functionalization of the drug deferasirox.

Variation at the C5 position and N–OR substituent further highlights the versatility of the strategy. Spirocyclic and *gem*-disubstituted substrates undergo efficient ring expansion, whereas monosubstituted analogues fail to initiate the key solvolysis step required for rearrangement. Diverse alkyl, allyl, propargyl, and carbohydrate-derived substituents at the N–OR site are all tolerated, enabling access to structurally diverse heterocyclic architectures (Scheme 28b). Importantly, the methodology could be extended to previously unexplored 4-selenazolidinone substrates, providing access to six-membered Se,N-heterocycles under analogous conditions. This extension significantly broadens the heteroatom diversity accessible through the strategy and further demonstrates the generality of the underlying ring-expansion concept.

Mechanistic investigations, supported by control experiments and DFT calculations, indicate that HFIP plays a multifaceted role in promoting C–N bond cleavage, stabilizing reactive intermediates, and facilitating nitrogen transfer and ring expansion. The process proceeds under exceptionally mild conditions without the need for metals, acids, or additional additives, emphasizing the unique activating ability of HFIP in skeletal rearrangement chemistry.

**Scheme 29:**
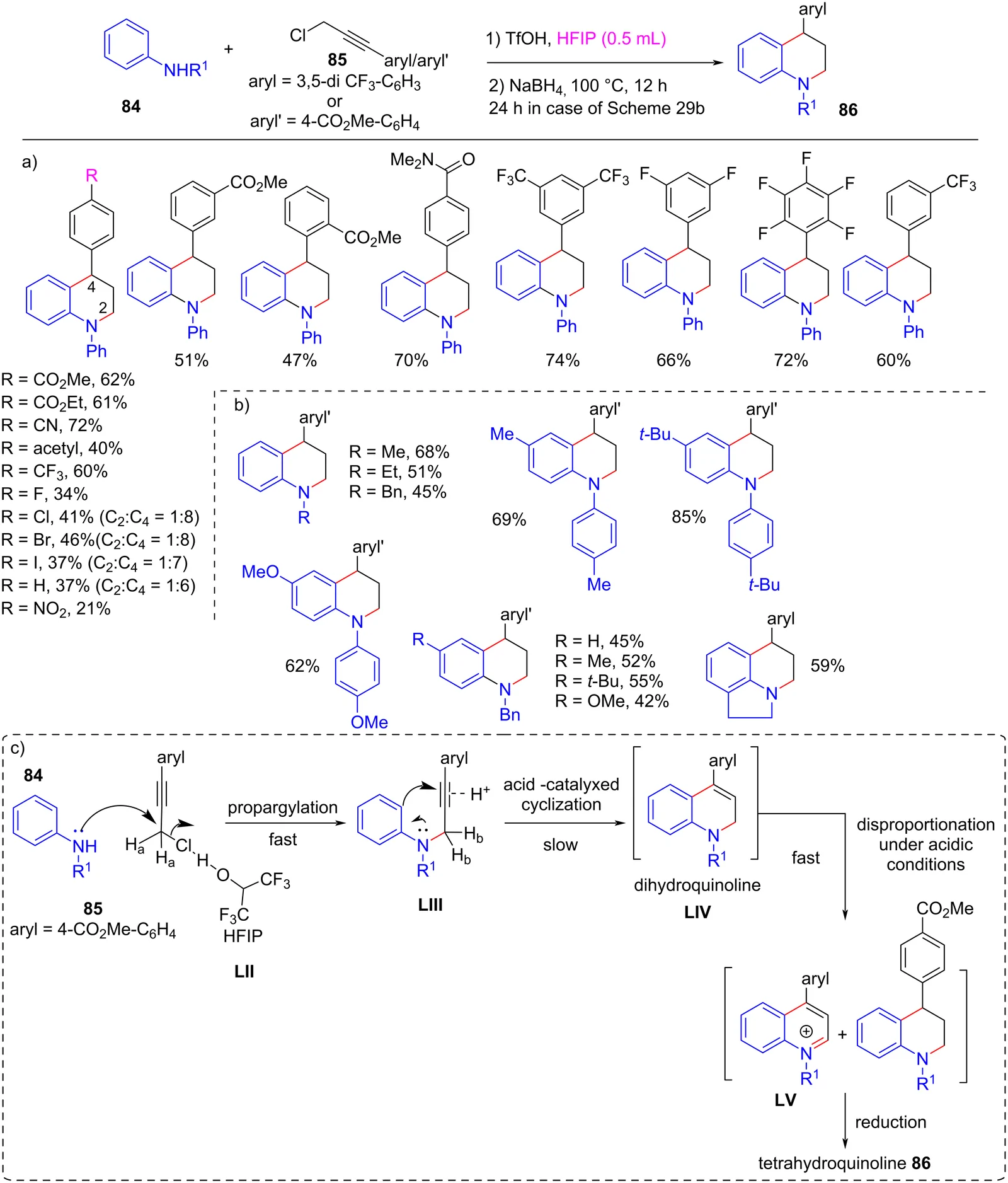
a) Reaction scope of propargylic chlorides. b) Scope of aniline moieties. c) Proposed mechanism.

**Six-membered fused saturated heterocycle construction:***Synthesis of C4-aryl-substituted tetrahydroquinolines:* In particular, HFIP-assisted activation of C–H and C-heteroatom bonds has enabled the development of efficient transition-metal-free heterocycle syntheses under mild reaction conditions [79]. A notable example is the one-pot synthesis of C4-aryl-substituted tetrahydroquinolines **86** from simple anilines **84** and propargylic chlorides **85** under transition-metal-free conditions and this protocol was developed by Chi and Lee (Scheme 29) [80]. In this transformation, HFIP plays a decisive role in activating the C–Cl bond of propargylic chlorides, thereby enabling propargylation of anilines even in acidic media. Subsequent acid-promoted cyclization and reductive rearomatization furnished tetrahydroquinoline products **86** efficiently through a sequential propargylation–cyclization pathway. This strategy offers operational simplicity, avoidance of metal catalysts, and broad substrate compatibility. This HFIP-enabled strategy highlights the broader synthetic potential of fluorinated alcohols in heterocycle construction. Compared with traditional metal-catalyzed tetrahydroquinoline syntheses, the protocol provides a more sustainable and operationally straightforward alternative while maintaining broad functional-group tolerance and scalability. At the same time, the dependence on substrate electronics and the moderate efficiency observed for certain electron-deficient systems illustrate the continuing challenges associated with fully general HFIP-mediated activation processes.

The methodology demonstrated substantial tolerance toward electronically diverse propargylic chlorides, including ester, amide, nitrile, ketone, trifluoromethyl, trifluoromethoxy, triflate, and halogen-substituted systems, generally affording moderate to good yields (Scheme 29a). Electron-deficient fluorinated substrates often provided improved reactivity, suggesting beneficial interactions between substrate electronics and HFIP-mediated activation. In contrast, strongly electron-withdrawing nitro substituents significantly reduced efficiency, likely because of competing hydrogen-bond interactions that interfere with effective substrate activation. Halogenated substrates, including chloro, bromo, and iodo derivatives, were also compatible, although regioisomeric mixtures were occasionally observed.

Variation of the aniline component further highlighted the scope and limitations of the protocol. Alkyl-, alkoxy-, and sterically substituted diarylamines underwent smooth cyclization to provide functionalized tetrahydroquinolines in moderate to excellent yields, whereas highly electron-deficient diarylamines proved less effective. The methodology also accommodated benzyl-protected anilines and heterocyclic substrates such as indoline, demonstrating applicability toward biologically relevant tetrahydroquinoline frameworks and late-stage scaffold diversification (Scheme 29b).

Mechanistic studies supported a pathway involving HFIP-assisted activation of the propargylic chloride **85**, followed by rapid formation of propargylic amine intermediates **LII** and **LIII** (Scheme 29c). Acid-catalyzed intramolecular cyclization generated dihydroquinoline intermediates **LIV**, which subsequently underwent disproportionation and reductive conversion of **LV** to the final tetrahydroquinolines **86**. Importantly, HFIP was proposed not only to activate the C–Cl bond but also to modulate the reactivity of sodium borohydride through hydrogen-bonded ionic complexes, thereby enabling compatibility with sensitive carbonyl-containing substrates under otherwise strongly reducing conditions.

**Scheme 30:**
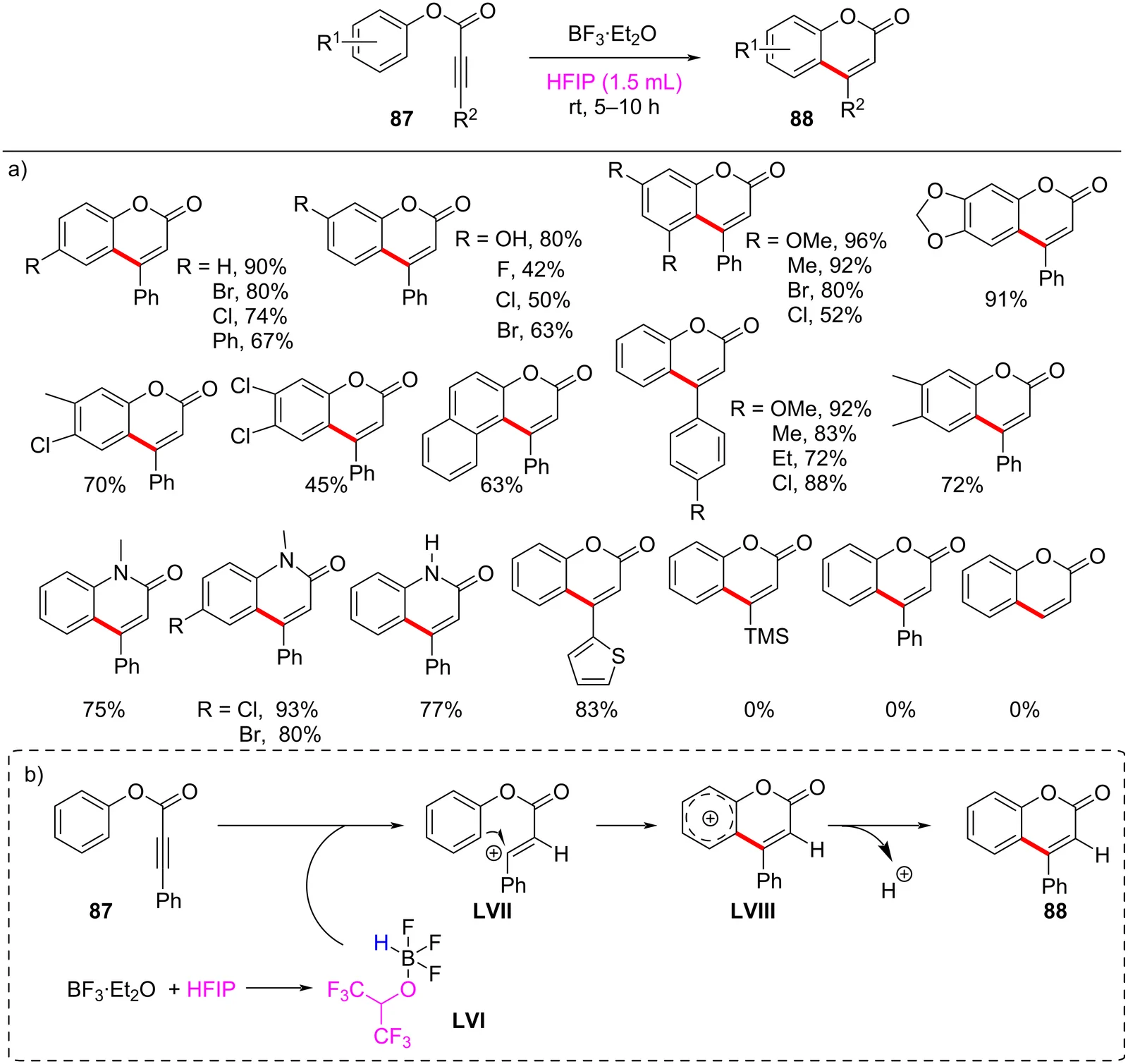
a) Substrate scope coumarin synthesis. b) Proposed mechanism.

*Intramolecular alkyne cyclization to coumarins:* Coumarins represent an important class of naturally occurring heterocycles with broad pharmacological relevance, including antioxidant, anticancer, antimicrobial, and neuroprotective activities [81]. In this context, HFIP-based catalytic systems have recently enabled efficient and sustainable approaches to coumarin construction, particularly through intramolecular alkyne cyclization strategies that proceed under metal-free and mild conditions [82]. Among these, a cooperative HFIP/BF_3_·Et_2_O system has emerged as a particularly effective platform, illustrating how solvent-catalyst synergy can be exploited to access complex oxygen heterocycles and this was reported by Wu and co-workers (Scheme 30) [82].

In this methodology, electron-rich and moderately substituted phenyl propiolates **87** undergo smooth cyclization to furnish 4-aryl- and heteroarylcoumarins **88** in generally high yields, whereas electron-withdrawing substituents tend to decrease reactivity, consistent with an electrophilic addition–cyclization mechanism (Scheme 30a). This contrasts with more electronically tolerant HFIP-only or radical-based systems, highlighting the greater charge-dependence of BF_3_-assisted pathways. Nevertheless, the protocol shows broad functional-group compatibility overall, including cyclic, heteroaromatic, and amide-derived substrates, with the latter significantly expanding access to nitrogen-containing analogues. This strategy avoids metal residues while maintaining operational simplicity, though it can require elevated temperatures for less reactive substrates.

Mechanistically, the reaction is proposed to proceed through in situ formation of a strongly acidic species **LVI** arising from interaction between HFIP and BF_3_·Et_2_O. This activated complex promotes carbocation formation **LVII** from the alkyne precursor **87**, followed by intramolecular cyclization (**LVIII**) to construct the coumarin framework **88**. HFIP plays a dual and non-trivial role: beyond serving as a polar, hydrogen-bonding medium, it stabilizes key cationic intermediates through an extended hydrogen-bond network and facilitates smooth progression of the electrophilic cascade (Scheme 30b). Compared with electrochemical or radical HFIP-enabled systems, this approach is more ionically driven, relying on carbocation stabilization rather than radical generation or redox processes.

This strategy is distinct in its reliance on electrophilic activation of alkynes rather than radical or oxidative pathways. Despite some limitations in substrate scope for deactivated systems, the methodology provides a valuable, scalable, and metal-free route to biologically relevant coumarin frameworks and further underscores the multifunctional role of HFIP as both solvent and catalytic partner in modern heterocycle synthesis.

**Scheme 31:**
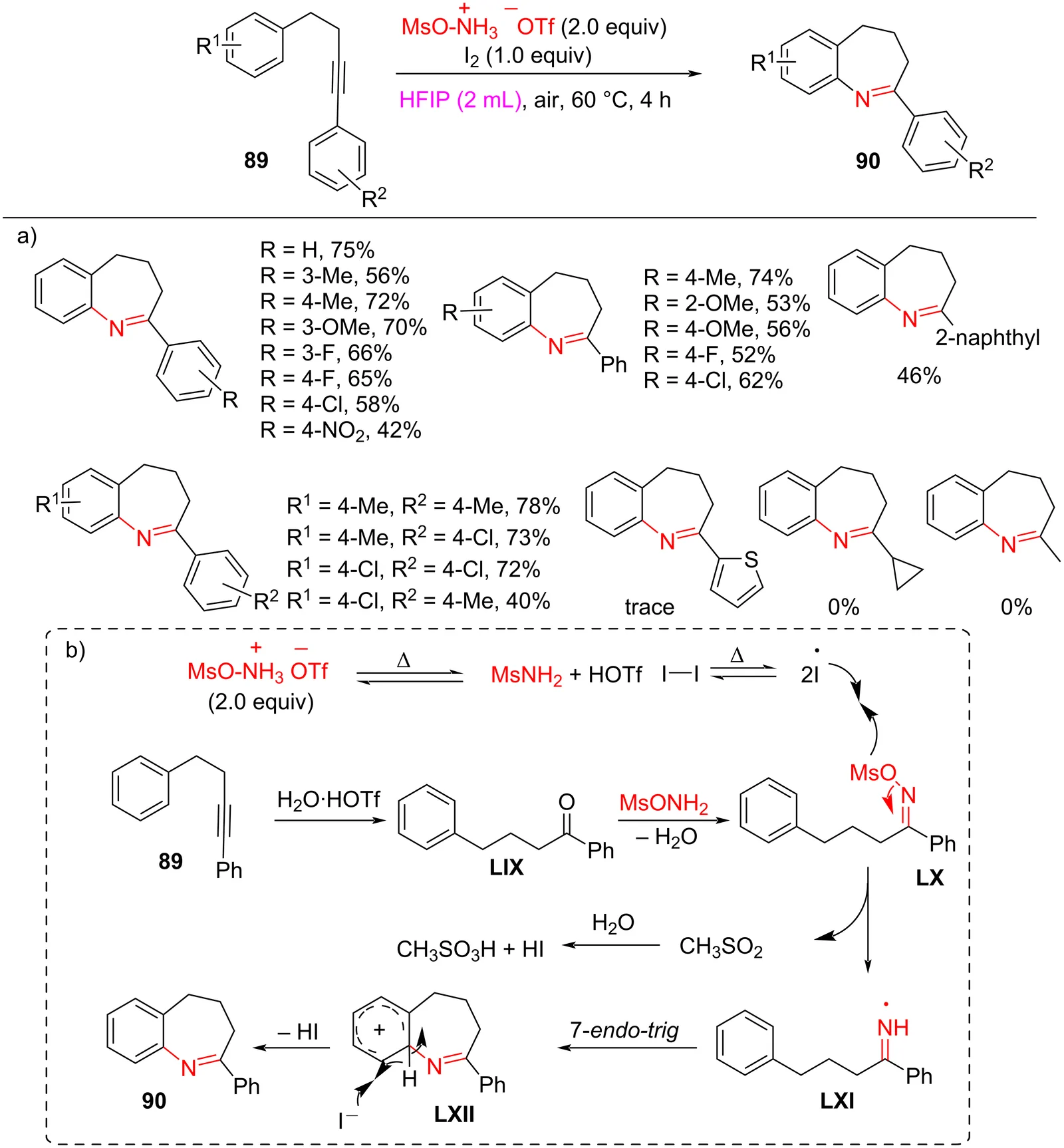
a) Scope of 1,4-diarylalkynes for the synthesis of **90**. b) Proposed mechanism.

**Seven-membered fused saturated heterocycle construction:***Direct synthesis of benzo[b]azepines:* Benzo[*b*]azepines **90** are structurally important seven-membered nitrogen heterocycles frequently found in bioactive molecules and drug candidates [83]. In this context, a metal-free I_2_-mediated [6 + 1] annulation using protonated aminating reagent (MsONH_3_OTf) and alkynes **89** has been developed by Yan and co-workers as an efficient route to this scaffold under mild conditions, highlighting the growing utility of radical and Brønsted acid cooperative systems in medium-ring heterocycle construction (Scheme 31) [84].

The method shows broad tolerance toward both electron-donating and electron-withdrawing substituents on the aromatic ring (Scheme 31a), with generally moderate to good yields across *para*-, *meta*-, and *ortho*-substituted substrates. The transformation displays relatively low sensitivity to electronic effects and steric variation, indicating a robust reaction manifold. However, limitations are observed with certain alkynes bearing heteroaryl (e.g., thienyl), strained alkyl (cyclopropyl), or simple alkyl substituents, which fail to deliver the desired products, suggesting constraints in radical stabilization and intermediate reactivity.

Mechanistically, the reaction is proposed to proceed through in situ generation of HOTf, which catalyzes alkyne hydration to yield **LIX** followed by condensation with MsONH_2_ to form an oxime intermediate **LX**. This is then activated by iodine to generate an N-centered radical **LXI** via N–O bond homolysis, followed by radical cyclization (**LXII**) to construct the seven-membered ring **90** (Scheme 31b). Compared with HFIP-stabilized ionic pathways or electrochemical radical systems, this transformation relies on a hybrid acid-radical sequence where iodine serves as the key redox mediator rather than an external oxidant or electrode. The absence of transition metals and the in situ generation of reactive species make the protocol operationally simple, although dependence on radical propagation and substrate compatibility limits its universality compared to more general HFIP-enabled cyclizations.

Overall, this benzo[*b*]azepine **90** synthesis complements other modern heterocycle-forming strategies by occupying a mechanistic space between ionic HFIP as a solvent mediated cyclizations and electrochemical radical processes. While HFIP-based systems excel in stabilizing cationic intermediates and electrochemical methods offer controlled redox activation, this iodine-mediated approach provides a practical alternative for accessing medium-sized N-heterocycles **90** through radical annulation under mild, metal-free conditions.

**Scheme 32:**
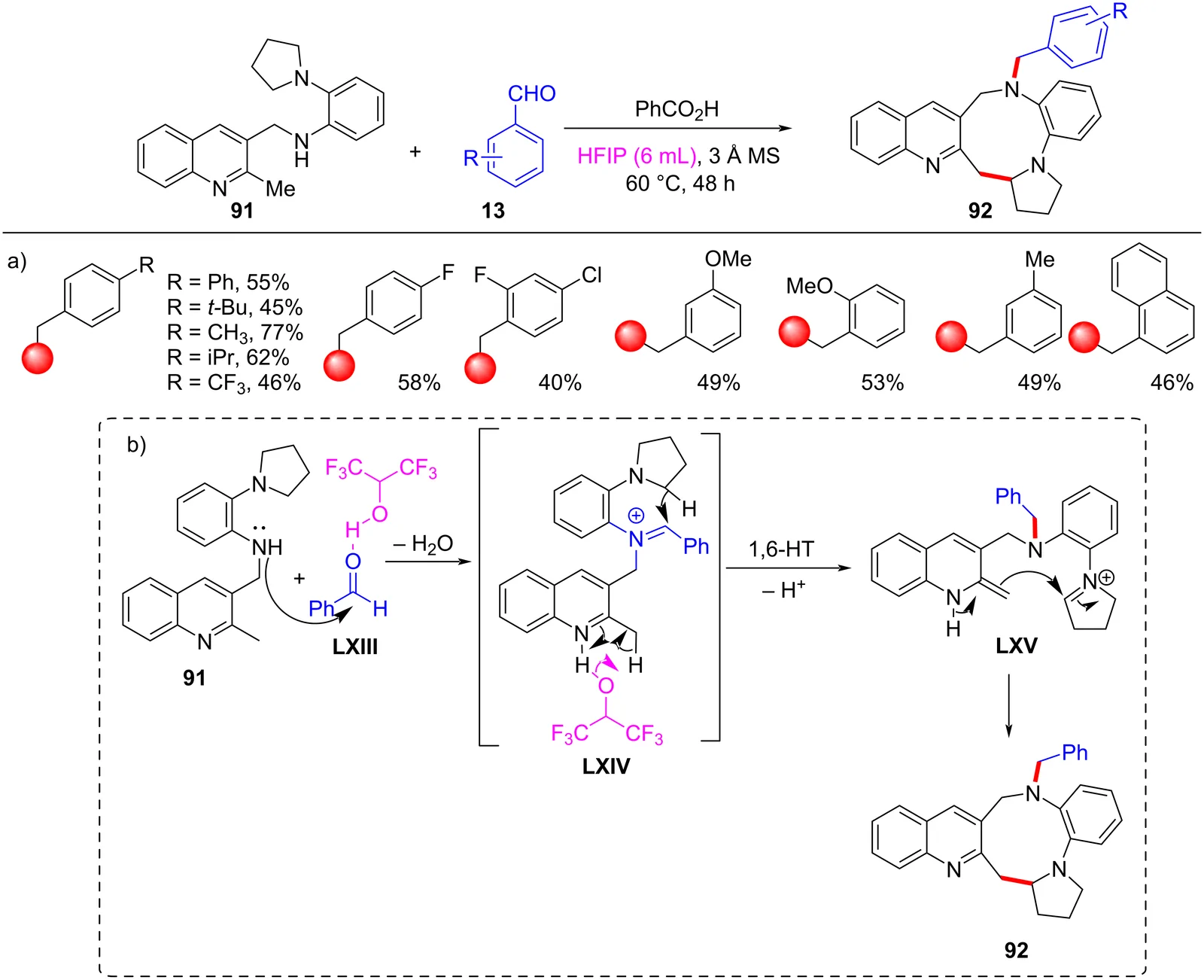
a) Substrate scope for the synthesis of nine-membered rings. b) Proposed mechanism.

**Nine-membered fused saturated heterocycle construction:***Synthesis of nine-membered heterocyclic derivatives:* Medium-sized ring systems, particularly nine-membered frameworks, are prevalent in complex natural products and bioactive molecules, yet their synthesis remains challenging due to unfavorable entropic constraints and transannular strain effects [85]. In response, a metal-free, redox-neutral hydride-transfer (HT)-triggered cross-dehydrogenative coupling (CDC) strategy has been developed by Xiao and co-workers for the construction of quinoline-fused nine-membered rings **92**, offering a step- and atom-economical alternative to traditional prefunctionalized approaches (Scheme 32) [86].

This transformation proceeds efficiently across a broad range of aromatic aldehydes and diamines, demonstrating generally good functional-group tolerance (Scheme 32a). Electron-rich aldehydes tend to give higher yields, whereas electron-withdrawing substituents lead to reduced efficiency, reflecting the key role of iminium ion stability in governing reactivity. Steric effects are also significant, with *meta*-substitution resulting in diminished yields, while heteroaromatic systems such as naphthaldehydes remain competent substrates. This CDC process is more strongly governed by iminium ion formation and hydride-transfer dynamics, making it particularly sensitive to electronic effects. While highly efficient and environmentally favorable, its strong dependence on iminium stability and electronic effects contrasts with the broader functional tolerance often observed in HFIP-stabilized radical or electrochemical systems. Nevertheless, it represents a valuable complement within modern CDC and medium-ring synthetic methodologies, particularly for accessing complex quinoline-fused architectures in a single-step, green fashion.

Mechanistically, HFIP plays a central dual role: it facilitates aldimine formation to generate iminium ion intermediates and stabilizes charged transition states through an extended hydrogen-bonding network (Scheme 32b). The reaction is initiated by HFIP-assisted iminium formation (**LXIII**), followed by an intramolecular hydride transfer that activates a C(sp^3^)–H bond and generates a second iminium species **LXIV**. Subsequent nucleophilic capture by an enamine intermediate **LXV** delivers the cyclized product **92**. The addition of benzoic acid further enhances acidity through a “combined acid” effect, reinforcing the catalytic environment. Compared with electrochemical or radical CDC methodologies, this approach is distinctive in its purely redox-neutral nature, avoiding external oxidants or catalysts while relying instead on internal hydride transfer and acid-assisted activation.

**Scheme 33:**
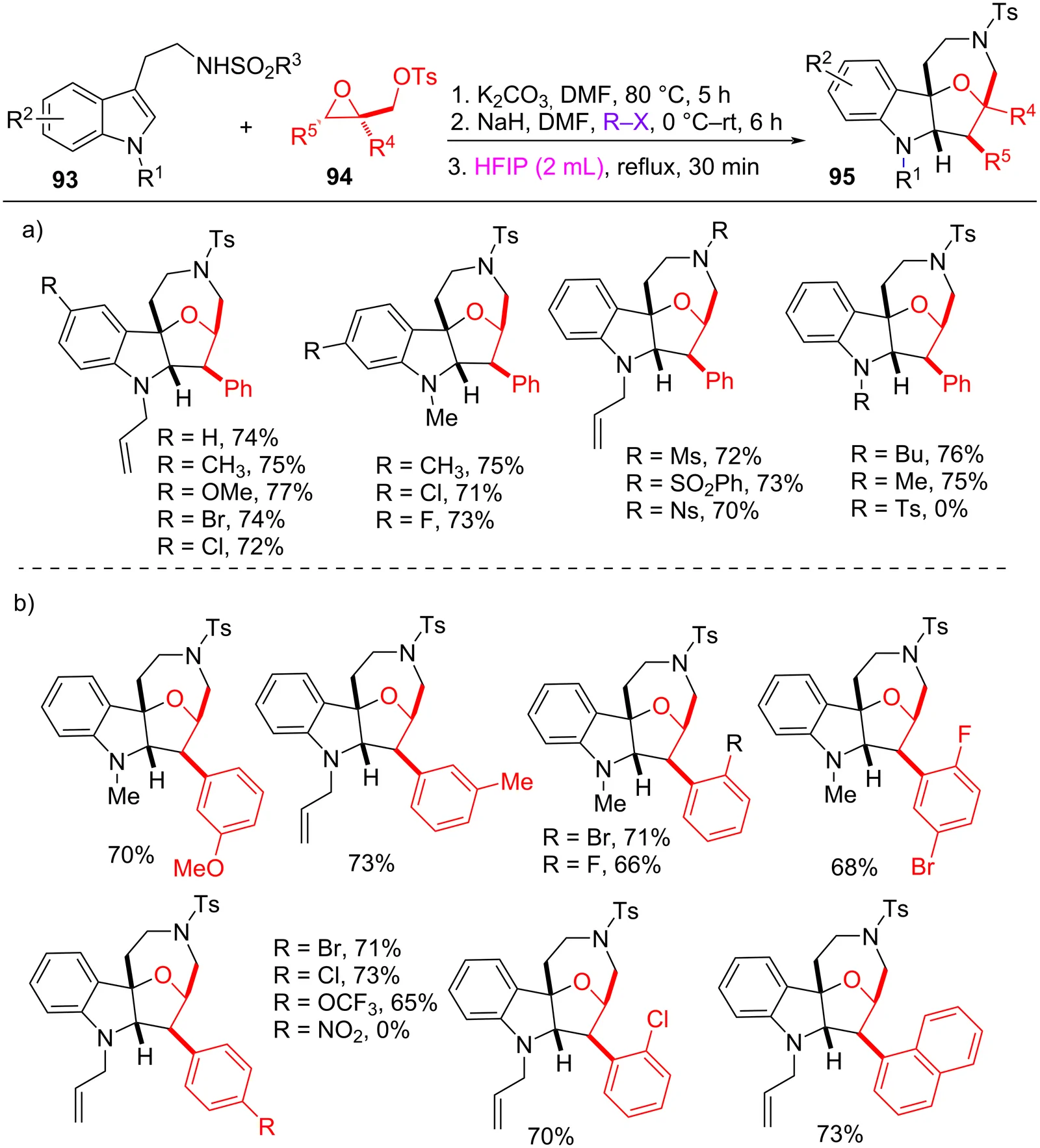
Substarte scope of (a) tryptamine and (b) epoxy tosylates derivatives.

**Polyfused saturated heterocycle construction:***Formal umpolung reactivity of indole derivatives:* Indole functionalization, particularly at the C2 and C3 positions, has long been dominated by its inherent nucleophilicity toward electrophiles [87–88]. In this context, an HFIP as a solvent promoted cascade process developed by Das and Chouhan involving epoxide-tethered indoles provides an unusual and conceptually important deviation from conventional indole reactivity, enabling a formal C3 umpolung through a dearomative cyclization–rearrangement sequence [89]. This strategy delivers highly complex fused-bridged indoline architectures **95** in a single operation with excellent regio- and diastereocontrol, significantly expanding the synthetic landscape of indole chemistry (Scheme 33).

The transformation exhibits a broad substrate scope across tryptamine derivatives **93** bearing diverse electronic and steric substitution patterns on the aromatic ring, including electron-donating and moderately electron-withdrawing groups, which generally furnish products **95** in high yields (Scheme 33). Variations in *N*-protection and *N*-alkyl substituents are also well tolerated, indicating that the reaction is relatively insensitive to modification of the indole side chain. However, clear limitations emerge when the intrinsic nucleophilicity of the indole C3 position is reduced, particularly under strongly electron-withdrawing *N*-substitution, or when the epoxide framework lacks sufficient π-activation, leading to unreactive or decomposed substrates. Compared with HFIP-stabilized ionic cyclizations or electrochemical radical processes, this system is more sensitive to subtle electronic tuning of the indole core and the epoxide electrophile.

Mechanistically, HFIP plays a decisive role in enabling the initial dearomative epoxide-indole cyclization by stabilizing developing cationic character through extensive hydrogen-bonding interactions. This activation triggers nucleophilic attack of the indole onto the epoxide, followed by an interrupted Plancher-type rearrangement that reorganizes the intermediate into a fused-bridged indoline framework. The process effectively reverses the conventional polarity of indole reactivity at C_3_, achieving a formal umpolung through substrate activation and rearrangement rather than prefunctionalization or external redox control. Compared with electrochemical or radical indole functionalizations, this approach uniquely combines dearomatization and skeletal rearrangement in a single cascade, relying primarily on HFIP-mediated ion stabilization rather than radical generation or metal catalysis.

Overall, this HFIP-enabled cascade represents a powerful example of complexity-building indole chemistry, converting simple indole-epoxide precursors into highly strained bridged frameworks with excellent selectivity. While its efficiency and stereocontrol are notable, its dependence on epoxide activation and indole electronic properties defines a narrower reactivity window compared to more general HFIP-assisted cyclizations. Nevertheless, it provides a distinct and valuable strategy for accessing architecturally complex indoline scaffolds and demonstrates how HFIP can unlock non-intuitive reactivity patterns in heteroaromatic systems.

## Conclusion and Outlook

In conclusion, HFIP has evolved from a specialized fluorinated solvent into a powerful reaction-enabling medium that plays a central role in modern heterocyclic chemistry. Its unique combination of strong hydrogen-bond-donating ability, high ionizing power, low nucleophilicity, and exceptional capacity to stabilize ionic and radical intermediates has enabled numerous synthetic transformations that are often difficult to achieve under conventional conditions. As highlighted throughout this review, HFIP not only serves as a solvent but frequently acts as a promoter, cocatalyst, proton-transfer mediator, and reaction-directing component, significantly influencing reaction pathways, selectivity, and overall efficiency.

The recent advances summarized from 2022 to 2025 clearly demonstrate the versatility of HFIP across a broad spectrum of heterocyclic ring constructions, including simple heterocycles, fused and polycyclic systems, spirocyclic frameworks, and saturated or non-aromatic heterocycles. HFIP-enabled methodologies have facilitated diverse transformations such as cyclizations, annulations, dearomatizations, rearrangements, electrochemical reactions, and cross-dehydrogenative coupling processes under mild, metal-free, oxidant-free, or redox-neutral conditions. In many cases, these strategies exhibit broad substrate scope, high chemo-, regio-, and stereoselectivity, operational simplicity, and improved sustainability compared with traditional protocols.

Mechanistically, the ability of HFIP to stabilize reactive intermediates through extensive hydrogen-bonding networks and cooperative interactions with catalysts or reagents has emerged as a recurring theme across many transformations. The solvent has proven particularly valuable in promoting cationic, radical, and cascade processes, thereby enabling rapid molecular complexity generation and access to structurally diverse heterocyclic scaffolds with significant pharmaceutical and synthetic relevance. At the same time, the reviewed studies also reveal certain limitations, including substrate-dependent reactivity, solvent cost, and challenges associated with solvent recovery and large-scale applications.

Nevertheless, the rapidly growing number of HFIP-mediated methodologies underscores its continuing impact on synthetic design and reaction development. Future progress in recyclable fluorinated solvent systems, asymmetric variants, electrochemical processes, photocatalytic transformations, and sustainable catalytic protocols is expected to further expand the utility of HFIP in heterocyclic synthesis. The authors have anticipated that the mechanistic insights and synthetic strategies discussed in this review will provide valuable guidance for the development of next-generation HFIP-enabled transformations and inspire further innovation in sustainable heterocyclic chemistry.

## Emerging Alternatives to HFIP

Although HFIP remains a valuable solvent in heterocyclic synthesis, its environmental, toxicological, and economic concerns have encouraged the search for more sustainable alternatives. Emerging approaches include alternative fluorinated alcohols, HFIP–water or mixed-solvent systems, and non-fluorinated hydrogen-bond-donating solvents, ionic liquids, and deep eutectic solvents. Solvent-free and low-solvent approaches, including mechanochemistry, electrochemistry, and photochemistry, also offer promising directions. Rather than seeking a single replacement for HFIP, future studies may focus on identifying reaction media that reproduce its specific beneficial properties while reducing its environmental impact. Comparative studies and solvent-recycling strategies will be important in assessing the practical potential of these alternatives.

## Data Availability

Data sharing is not applicable as no new data was generated or analyzed in this study.
